# Mucin‐Inspired Filamentous Sulfated Copolymers Effectively Inhibit Human Respiratory Syncytial Virus (hRSV) Infectivity

**DOI:** 10.1002/advs.202515908

**Published:** 2025-12-01

**Authors:** Raju Bej, Enyu Xie, Kai Ludwig, Robert F. Schmidt, Yannic Kerkhoff, Robert Dalgliesh, Nilanjan Paul, Michael Gradzielski, Andreas Herrmann, Christian Sieben, Rainer Haag

**Affiliations:** ^1^ Jyoti and Bhupat Mehta School of Health Sciences and Technology Indian Institute of Technology Guwahati Guwahati 781039 India; ^2^ Nanoscale Infection Biology Group Helmholtz Centre for Infection Research Inhoffenstraße 7 38124 Braunschweig Germany; ^3^ Institut für Chemie und Biochemie Freie Universität Berlin Takustraße 3 14195 Berlin Germany; ^4^ Stranski‐Laboratorium für Physikalische und Theoretische Chemie Institut für Chemie Technische Universität Berlin 10623 Berlin Germany; ^5^ IT and Data Services Zuse Institute Berlin Takustraße 7 14195 Berlin Germany; ^6^ ISIS Pulsed Neutron and Muon Source Science and Technology Facilities Council, Rutherford Appleton Laboratory Harwell Oxford Didcot OX11 0QX UK; ^7^ Institute for Genetics Technische Universität Braunschweig Spielmannstr. 7 38106 Braunschweig Germany

**Keywords:** biocompatible, cryo‐EM, filamentous structure, hRSV inhibition and virucidality, mucin‐inspired copolymer, RAFT polymerization, small angle neutron scattering (SANS)

## Abstract

Virucidal compounds capable of binding to and disrupting viruses represent a promising avenue for antiviral applications. In this study, the development of high molecular weight (≈300 kDa) dendronized polyglycerol‐based mucin‐inspired amphiphilic statistical copolymers (MIACPs) is reported using the RAFT polymerization technique. These copolymers comprise ≈30% repeat units containing aliphatic C11 carbon chains with terminal carboxylate (MIACP‐1) and alkyl (MIACP‐2) functionalities, while the remaining ≈70% of the repeat units consist of dendronized polyglycerol sulfates. Structural characterization using cryo‐electron microscopy (cryo‐EM) and small‐angle neutron scattering (SANS) reveals that **MIACPs** form single‐chain filamentous structures, similar to natural porcine gastric mucin (PGM). These biocompatible **MIACPs** exhibit strong, sulfate‐dependent inhibition of human respiratory syncytial virus (hRSV), with exceptionally low IC_50_ values (C = ≈0.25 µg mL^−1^). The virucidal activity is assessed using serial dilution experiments, which confirms that **MIACPs** demonstrate virucidal activity, indicating a very strong binding affinity of the polymers to the hRSV. In contrast, a similar molecular weight homopolymer composed solely of sulfated dendronized repeat units exhibits comparable hRSV inhibition activity but lacks any virucidal effect. Therefore, designing a statistical copolymer with ≈30% virucidal functionality is unique in that it renders the copolymer virucidal without compromising its inhibitory activity.

## Graphical abstract

Virucidal polymers serve as effective antivirals by not only inhibiting viruses but also destroying them. Inspired by the chemical properties of mucin‐mimetics, dendronized polyglycerol‐sulfated, mucin‐inspired amphiphilic copolymers (MIACPs) have been developed. These MIACPs form mucin‐like elongated fibers, as confirmed by cryo‐electron microscopy and small‐angle neutron scattering. MIACPs demonstrate strong potential in preventing infections caused by human respiratory viruses.

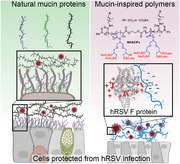

## 1 Introduction

Human respiratory syncytial virus (hRSV) is a leading cause of acute lower respiratory tract infections in infants, as well as in elderly individuals and high‐risk adults with chronic lung condition or a compromised immune system.^[^ ^1^, ^2^, ^3^ ^]^ Despite its clinical significance,^[^ ^4^ ^]^ there remains a lack of effective vaccines^[^ ^5^ ^]^ or widely available antiviral treatments for hRSV, highlighting the urgent need for new treatment options. As a result, the development of broad‐spectrum, potent, and biocompatible antiviral inhibitors remains a critical research priority.

Preventing hRSV infection at its early stages represents an alternative strategy to inhibit viral progression. hRSV initiates infection by interacting with cell surface heparan sulfate proteoglycans (HSPGs) or other sulfated proteoglycans.^[^ ^6^, ^7^ ^]^ Additionally, several membrane proteins have been implicated in facilitating hRSV entry into host cells.^[^ ^8^ ^]^ On the viral side, the envelope glycoprotein (G protein) and fusion protein (F protein) mediate attachment to the target cell membrane and promote membrane fusion.^[^ ^9^, ^10^ ^]^ At the molecular level, the negatively charged sulfate or carboxyl groups of HSPGs or heparin bind to clusters of positively charged basic amino acids within the linear heparin‐binding domain (HBD) of hRSV glycoproteins.^[^ ^11^ ^]^ This HSPG‐HBD interaction is a common and critical feature in hRSV‐mediated infection, making it an attractive target for the development of negatively charged antiviral agents.

In this context, heparan sulfate‐mimetic polymers and peptides have emerged as promising inhibitors of hRSV due to their ability to bind viral glycoproteins.^[^ ^12^ ^]^ Inspired by these findings, our group explored heparin‐mimetic linear and hyperbranched polyglycerol sulfates, which showed strong potential as antiviral agents owing to their biocompatibility.^[^ ^13^ ^]^ These results further motivated us to design functional polyglycerol sulfates with varied molecular weights, architectures, and structural compositions, aiming to broaden the scope of polysulfate‐based inhibitors with enhanced anti‐hRSV activity.

Mucin, a high molecular weight glycoprotein, is a key component of the protective mucus layer.^[^ ^14^ ^]^ Inspired by its structure and function, various mucin‐inspired biomaterials have been developed, demonstrating excellent inhibitory activity against a range of pathogens.^[^ ^15^, ^16^, ^17^, ^18^, ^19^ ^]^ This effectiveness is attributed to their unique characteristics, including high molecular weight, filamentous structure, and strong electronegative charge. Recently, our group developed a series of mucin‐inspired dendronized polyglycerol sulfates with varying molecular weights and found them to be potent inhibitors of sulfate‐binding viruses such as HSV‐1, SARS‐CoV‐2, and their variants.^[^ ^20^, ^21^, ^22^ ^]^ In those studies, polymers with longer filamentous structures and higher overall electronegative charge emerged as the most effective inhibitors. Their antiviral activity was comparable to that of antibodies,^[^ ^23^ ^]^ making them among the most promising synthetic inhibitors reported to date. These findings motivated us to investigate functionalized, high molecular weight, filamentous polymeric architectures for the inhibition of hRSV.

**Scheme 1:**
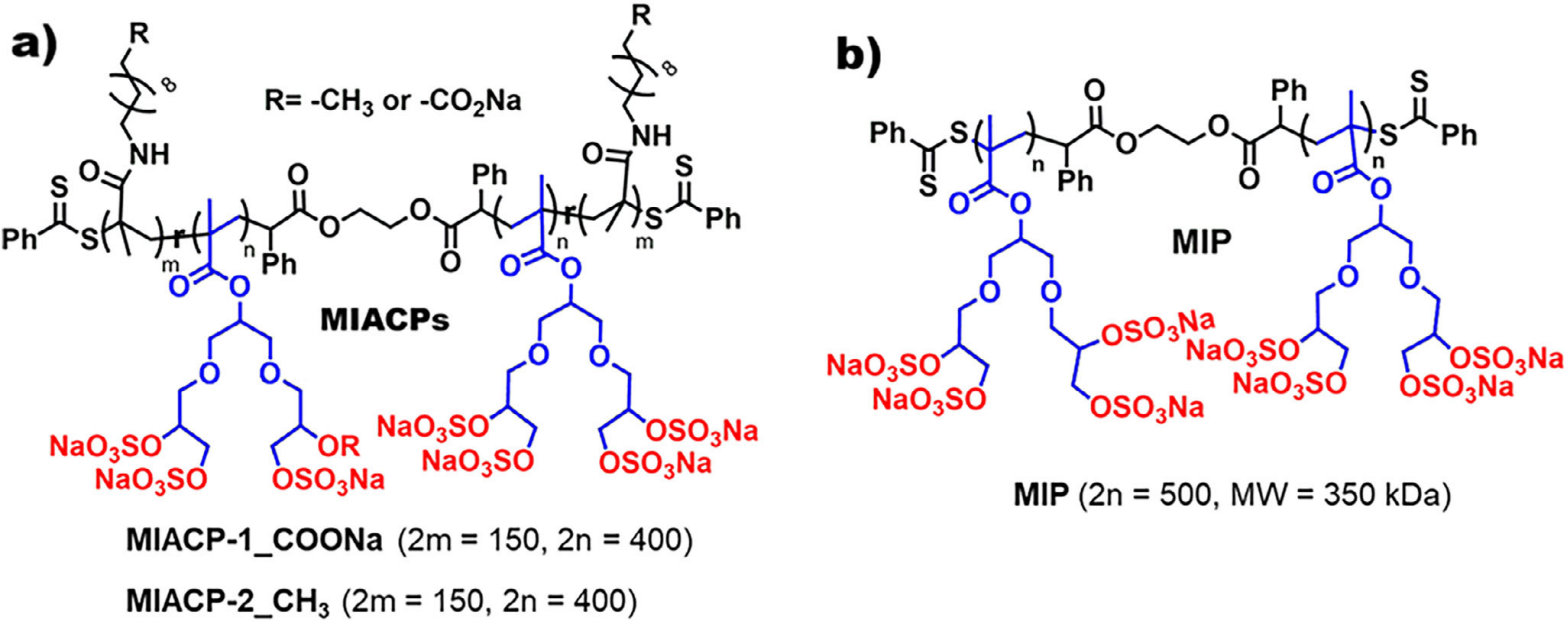
Chemical structure of the investigated mucin‐inspired a) amphiphilic copolymers (**MIACPs**) and b) polymer (**MIP**). **MIACPs** contain ≈30% repeat units of virucidal functionality: aliphatic C11 carbons chain with terminal carboxylate functional groups (**MIACP‐1**), alkyl functional group (**MIACP‐2**). All cases remaining ≈70% repeat units’ functionality from dendronized polyglycerol sulfates. **MIP** contains only dendronized polyglycerol sulfates as repeat units without any aliphatic functionality.

Another important consideration is that virus binding mediated by electrostatic interactions may be a reversible process, potentially allowing virus particles to detach from the inhibitors and proceed to infect host cells‐thereby limiting their therapeutic efficacy. This limitation can be addressed by employing inhibitors with virucidal properties that irreversibly deactivate viruses.^[^ ^24^ ^]^ This insight further motivated us to enhance the previously developed mucin‐inspired platform by introducing a defined percentage of virucidal functionality, forming the basis for the design of new copolymers. In selecting the virus‐disrupting moieties, alkyl chains with aliphatic C11 carbon lengths have been shown to irreversibly disrupt viral membranes through hydrophobic interactions,^[^ ^25^, ^26^, ^27^, ^28^ ^]^ which guided our development of amphiphilic copolymers (**Scheme** **1**a).

Amphiphilic polymers often tend to self‐assemble into various aggregated structures.^[^ ^29^, ^30^, ^31^, ^32^, ^33^ ^]^ However, in our research, we aimed to retain a filamentous morphology instead of promoting other ways of aggregation, in order to leverage the structural advantages of mucin‐like filaments.^[^ ^22^ ^]^ Previous studies have reported that other types of aggregation can be minimized by carefully tuning the hydrophilic‐hydrophobic balance of amphiphilic polymers. Notably, systems containing up to ≈30% hydrophobic units have been shown to be very positive in that aspect.^[^ ^34^, ^35^, ^36^, ^37^ ^]^ Guided by this, we designed amphiphilic copolymers composed of ≈70% dendronized sulfated groups and ≈30% C11 hydrophobic units (Scheme 1a). The sulfated groups are intended to inhibit viral binding via electrostatic interactions with positively charged regions on viral surface proteins, while the hydrophobic C11 domains are expected to provide virucidal activity by disrupting the viral membrane.

In this work, we present the synthesis and comprehensive characterization of mucin‐inspired amphiphilic copolymers (MIACPs) incorporating C11 aliphatic chains, along with their homopolymer counterpart (MIP) of similar molecular weight (Scheme 1b). We investigate their morphology in aqueous solution using cryo‐electron tomography (Cryo‐ET) and small‐angle neutron scattering (SANS), assess their biocompatibility, and evaluate their antiviral and virucidal efficacy against hRSV.

## 2 Results and Discussion

### 2.1 Synthesis and Characterization of Mucin‐Inspired Amphiphilic Copolymers (MIACPs)

The chemical structures of the investigated polymers are depicted in Scheme 1. We report three different dendronized polysulfates with similar molecular weights (**Table** **1**). The first group consists of copolymers (MIACPs) comprising two components: ≈70% repeat units of sulfated dendronized oligoglycerol methacrylate (OGMA), and ≈30% repeat units containing an aliphatic C11 carbon chain terminated with either a carboxylate group (**MIACP‐1**) or a purely aliphatic group (**MIACP‐2**). The second is a homopolymer (**MIP**), which lacks any hydrophobic functionality and serves as a control to assess the impact of virucidal groups in MIACPs. Due to their amphiphilic nature, **MIACPs** carry a risk of particle aggregation. To minimize this, hydrophilic and hydrophobic units were statistically distributed within the copolymer chains.^[^ ^38^ ^]^

**Table 1:** Characterization of investigated polymers.

Polymers	MW^a^	Elemental Analysis				DoF^b^	ζ [mV]^c^
		Carbon (C)	Hydrogen (H)	Nitrogen (N)	Sulfur (S)		
**MIACP‐0**	150	43.5	6.9	0.78	0.7	0	−9.0 ± 1.9
**MIACP‐1**	320	29.2	3.8	0.59	13.9	94	−51.2 ± 6.5
**MIACP‐2**	310	28.9	3.8	0.45	14.8	92	−47.2 ± 3.4
**MIP**	350	29.9	3.5	0.22	18.6	94	−56.2 ± 4.8

^a)^ ^1^H NMR determined molecular weight in kDa;

^b)^ DoF stands for the percentage of degree of functionalization (sulfates) of the total hydroxyl groups;

^c)^ Zeta potential (ζ) for the aqueous solution (C = 1.0 mg mL^−1^) of the polymers.

For synthesis, we first optimized the polymerization conditions to prepare a relatively high‐molecular‐weight precursor copolymer, **P1** (pOGMA‐co‐pNHSMA), on a gram scale (Scheme S1, Supporting Information). Synthesis and characterization of **P1** are provided in the Supporting Information. N‐Hydroxysuccinimide methacrylate (NHSMA) units were selected as pre‐functional groups, which can later be replaced by amine functionalities to install virucidal moieties.^[^ ^39^ ^]^ The composition of copolymer **P1** was determined by ^1^H NMR analysis (Figure S1, Supporting Information), which confirmed that ≈30% of the repeat units were NHSMA. Considering ≈80% conversion of individual monomers, the molecular weight of **P1** was estimated to be 183 kDa, closely matching the size exclusion chromatography (SEC) measurement (*M*_W_ = 128 kDa) (Figure S2, Supporting Information), indicating successful controlled radical copolymerization. The NHS units in **P1** were then substituted with either commercially available 11‐amino undecanoic acid or 11‐amino undecane via standard nucleophilic substitution, yielding **P2a** and **P2b**, respectively. The disappearance of the NHS peak at *δ* = 2.88 ppm and the appearance of additional peaks at *δ* = 1.35–0.85 ppm region (corresponding to ‐CH_2_ protons from the aliphatic chain) for **P2a** and **P2b** in the ^1^H NMR spectrum confirmed successful substitution (Figures S3 and S4, Supporting Information). The integration of aliphatic protons relative to glycerol units further supported ≈30% incorporation of hydrophobic C11 units.

The acetonide groups of **P2a** were deprotected to yield **P3a** which was then sulfated to produce **MIACP‐1** (Scheme S1, Supporting Information). The disappearance of the *δ* = 1.4–1.35 ppm proton peak in the ^1^H NMR spectrum of **MIACP‐0** (Figure S5, Supporting Information) confirmed deprotection. Additionally, the shift in the methylene proton peak adjacent to the sulfate groups in the ^1^H NMR spectrum of **MIACP‐1** (Figure S7, Supporting Information), compared to **P3a**, and confirmed the successful introduction of electronegative sulfate groups.

Elemental analysis of **MIACP‐1** (Table 1) showed >90% sulfation of the hydroxyl groups in **P3a**. The molecular weight of **MIACP‐1**, estimated from ^1^H NMR, was ≈320 kDa, matches with the molecular weight (*M*_W_ = 290 kDa) obtained from size exclusion chromatography (SEC) (Figure S8, Supporting Information) and within the molecular weight range for the large, glycosylated regions of mucin polymers.^[^ ^40^ ^]^

**MIACP‐2** was synthesized using similar reaction conditions as described for **MIACP‐1**. For that, the acetonide groups of **P2b** were deprotected to yield **P3b** (**MIACP‐0**) which was then sulfated to produce **MIACP‐2** (Scheme S1, Supporting Information). The characterization data are shown in Table 1 and in the Supporting Information (Figures S6, S9, and S10, Supporting Information). The investigated homopolymer, **MIP**, was synthesized using a previously reported protocol from the group, with slight modifications (Scheme S2, Supporting Information). Characterization data are provided in Table 1 and in the Supporting Information (Figures S11–S15, Supporting Information).

### 2.2 Morphology Investigation: Cryo‐EM and SANS Analysis

**Figure 1:**
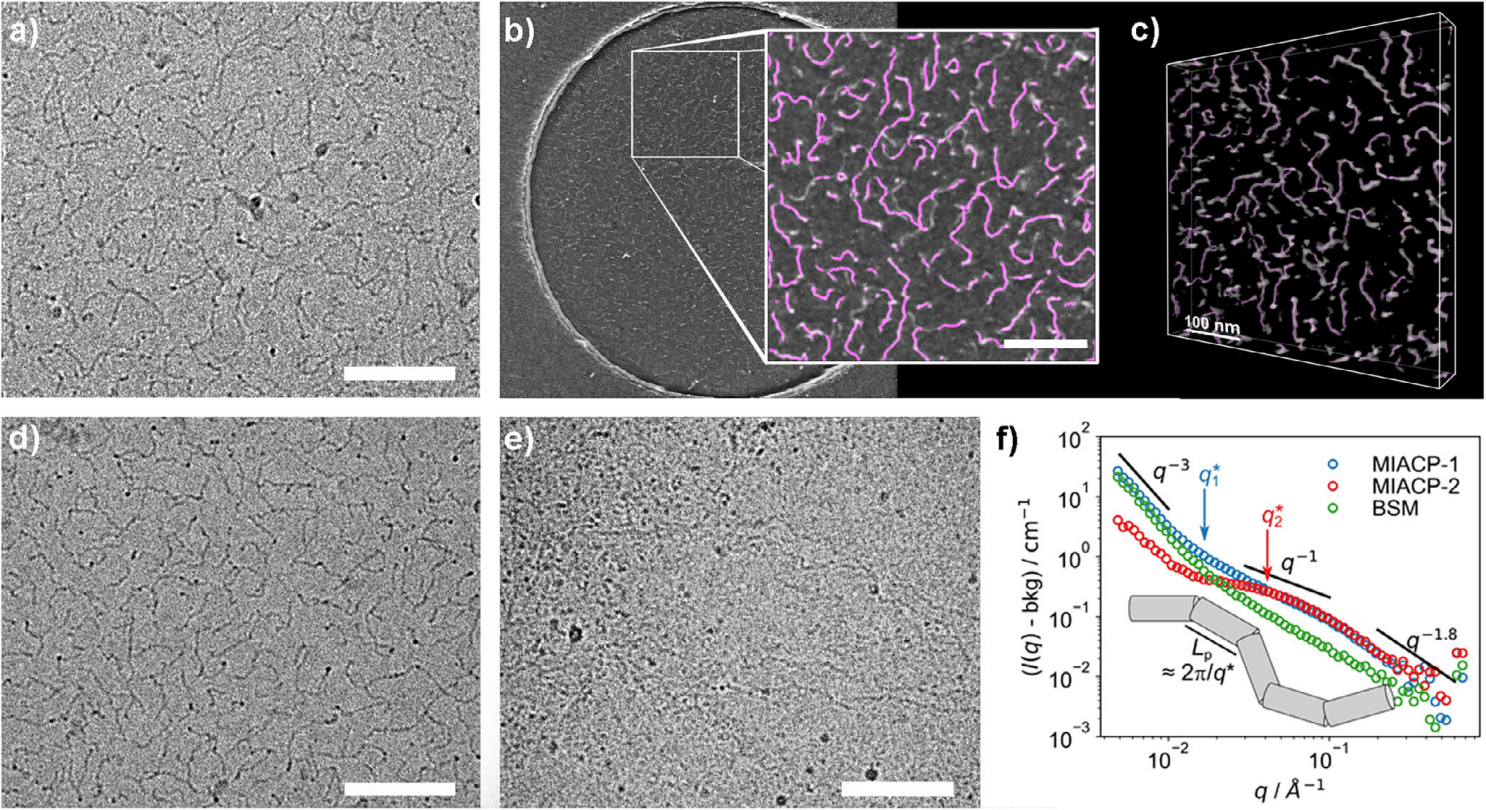
a) Cryo‐EM image and b) cryo‐electron tomogram of **MIACP‐1** in aqueous solution (C = 1.0 mg mL^−1^); the backbone structures determined with Fiji are highlighted in pink in the enlarged section. c) The 3D volume reconstructed from the cryo‐ET tilt image series (± 64°, 2° increments): Unlike 2D cryo‐EM images (= projections), this allows the determination of the actual 3D length of the amphiphilic elongated fibers. The average calculated length was 83.1 ± 29.2 nM. d), Cryo EM images of **MIACP‐2** and e) **PGM**, each in aqueous solution (C = 1.0 mg mL^−1^). All scale bars correspond to 100 nM. f) Small angle neutron scattering (SANS) spectra of **MIACP**s and BSM (C = 20 mg mL^−1^). A fiber structure should lead to a q^−1^ power law in mid‐q, which is more pronounced for **MIACP‐1**. The lower cut‐off of the q^−1^ power law region, q^*^, is inversely related to the persistence length of the fiber.

The overall electronegative charge of sulfated **MIACPs** and **MIP** was confirmed by their negative zeta potential values, attributed to the highly sulfated functionalities (Table 1). Cryo‐EM was measured to investigate the morphology of **MIACPs** in aqueous solution. Both **MIACP‐1** and **MIACP‐2** exhibited elongated filamentous structures (**Figure** **1**a,d). Cryo‐ET was employed to determine the 3D structure and length of the amphiphilic elongated fibers. To eliminate human bias, the fiber length distribution was semi‐automatically analyzed with Fiji^[^ ^41^ ^]^ using 3D skeleton analysis^[^ ^42^ ^]^ of pre‐processed cryo‐ET volume stacks (illustrated for **MIACP‐1** in Figure 1b,c and in the supporting movie SM‐01). Using this calculation, the average length for **MIACP‐1** and **MIACP‐2** were 83.1 ± 29.2 nM and 85.3 ± 19.0 nM, which is aligned well with the theoretical average stretched length of ≈150 nM of **MIACPs** (average number of repeat units = 500), calculated based on the atomic distance in the polymer chain. The observed length distribution of fibers is plausible considering the polydispersity (*Đ* = 1.5) in **MIACPs**.

Interestingly, ≈30% of the hydrophobic functional groups in the case of **MIACPs** did not affect the overall filamentous morphology of the copolymers. Since aliphatic C11 carbon chains are randomly distributed throughout the polymer backbone, the possibility of aggregation is reduced and filamentous structures are formed.

For comparison, the morphology of commercially available porcine gastric mucin (PGM), was also evaluated. It displayed thread‐like elongated fiber structures (Figure 1e), suggesting that **MIACPs** mimic the mucin‐like elongated morphology.^[^ ^43^ ^]^

Small angle neutron scattering (SANS) was further applied to evaluate the morphology of **MIACPs**. The SANS spectra of the **MIACPs** with subtracted background (cell and incoherent scattering) are shown in Figure 1f. The spectra can be divided into three regimes. At low *q*, where large structures are visible, the intensity roughly follows a *q* ^−3^ power law, which indicates the presence of large‐scale inhomogeneities with sizes > 10^2^ nM, i.e., clusters of polymers, as observed frequently in cross‐linked polymer gels^[^ ^44^, ^45^ ^]^ or in entangled polymer solutions.^[^ ^46^, ^47^ ^]^ The fact that such domains are seen in SANS, when there were none in cryo‐EM, can be explained by the much higher concentration (20 mg mL^−1^ for SANS as opposed to 1.0 mg mL^−1^ for cryo‐EM). The increased concentrations of the SANS measurements were chosen to ensure a sufficiently high scattering intensity. At such high concentrations, the polymers overlap and entangle, giving rise to large‐scale inhomogeneities visible in low‐q. At intermediate *q*, meaning on a more local scale, the intensity scales roughly as *q* ^−1^, as expected for a filamentous structure. Notably, the *q* ^−1^ region is significantly more pronounced for **MIACP‐1** and extends to lower *q* values. This suggests that the carboxylate moiety at the end of the hydrophobic chain promotes the formation of longer and stiffer fibers. In contrast, for **MIACP‐2,** one observes a markedly different scattering pattern at mid‐ and low‐q, which indicates the formation of more compacted structures on the length scale of 3‐5 nM. This may be attributed to the presence of the hydrophobic C_11_‐chains, whereas the **MIACP‐1** has a charged group at the end of this chain. At high *q*, the spectra of **MIACP‐1** and **MIACP‐2** look identical. On such small length scales, the local structure of the polymer chain is visible. The *q* ^−1.8^ scaling suggests that the polymer chains are slightly swollen, as expected for a polymer in a good solvent. For a comparison to mucin, we investigated BSM as a representative of commercially available natural mucin, using small‐angle neutron scattering (SANS). The resulting scattering pattern closely resembles that observed for **MIACP‐1**. Therefore, SANS data confirmed mucin‐like filamentous morphology for **MIACPs**.

### 2.3 Biocompatibility and Anticoagulant Assay

**Figure 2:**
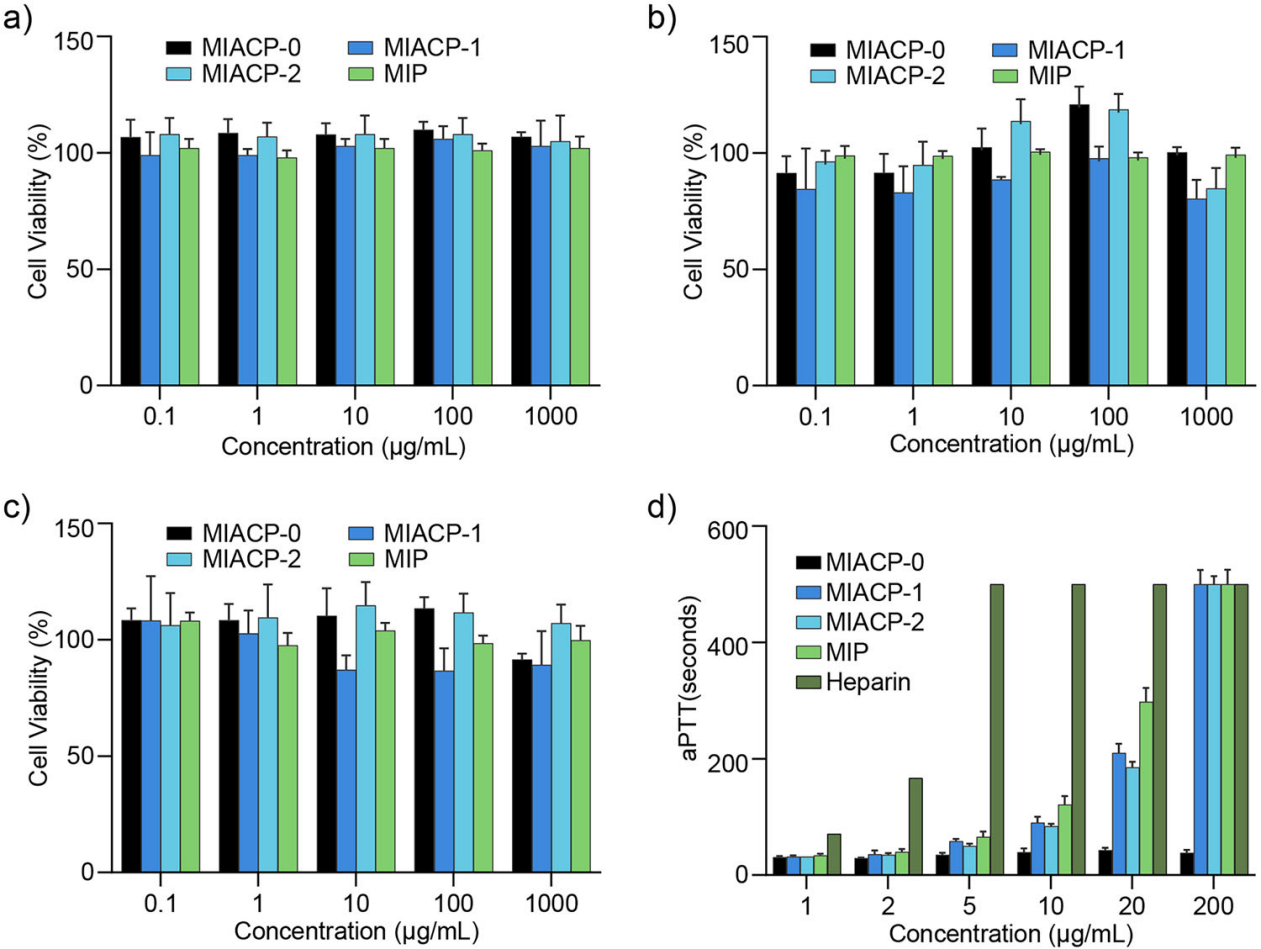
Cell viability of **MIACPs** and MIP in a) A549, b) HBE and c) Vero E6 cell line up to 1.0 mg mL^−1^ after 24 h of incubation. d) Activated partial thromboplastin time (aPTT) of plasma treated with **MIACP‐1** (dark blue), **MIACP‐2** (light blue), **MIP** (light green) at different concentrations. Heparin (dark green) and non‐sulfated MIACP, MIACP‐0 (black) were used as controls. Values are expressed as mean ±SD, *n* = 3.

The cytocompatibility of the **MIACPs** and **MIP** was evaluated on A549, HBE, and Vero E6 cell lines at polymer concentrations of up to 1.0 mg mL^−1^. The results suggest that **MIACPs** are biocompatible (**Figure** **2**a–c) in different cell lines and the introduction of C11 aliphatic functionality did not result in significant toxicity compared to the control polymer, **MIP**. The inherent anticoagulant activity of sulfated polymers generally limits their direct application.^[^ ^48^ ^]^ In this context, the **MIACPs** exhibited negligible anticoagulant activity up to 10 µg mL^−1^ compared to heparin (Figure 2d). Our studies further revealed that anticoagulant activity increased with the degree of sulfation, although it remained substantially lower than that of heparin.

### 2.4 Respiratory Syncytial Virus Inhibition and Virucidal Experiments

**Figure 3:**
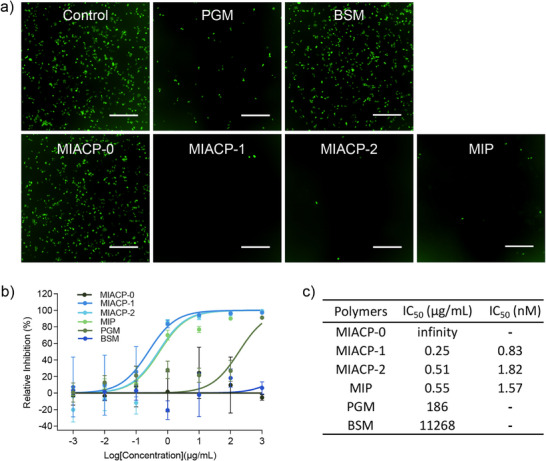
Activity of **MIACPs** against hRSV infection. hRSV‐GFP was mixed with compounds at indicated concentrations for 45 min at 37 °C before incubating with A549 cells for 2 h. Viruses mixed with double‐distilled water (ddH_2_O, the solvent of compounds) were used as the control group. The inoculum was then replaced with fresh medium. 24 h later, cells were imaged, and the number of infected cells (with GFP signal) was detected by Incucyte. **a)** Fluorescent images of cells infected by hRSV‐GFP treated with given compounds (1.0 mg mL^−1^). The green fluorescence observed in the infected cells is attributable to the expression of GFP; scale bar: 500 µm. b) Dose‐response curves of **MIACPs** and **MIP** against hRSV‐GFP (n = 3). Relative inhibition rates were expressed relative to the number of infected cells detected in the control group. The error bars indicate mean ± SEM. c) Table with the IC_50_ values for the hRSV inhibition of the investigated copolymers. Commercially available natural mucins (BSM and PGM) and non‐sulfated MIACP, **MIACP‐0** were used as controls.

We investigated the antiviral activity of **MIACPs** and **MIP** against hRSV using an hRSV subtype A long strain containing a GFP reporter gene (hRSV‐GFP).^[^ ^49^ ^]^ A549 lung epithelial cells were used to evaluate the anti‐hRSV efficacy (**Figure** **3**). In an infection inhibition assay, the dose‐response curves demonstrated that both **MIACPs** and **MIP** inhibited hRSV‐GFP, whereas the non‐sulfated copolymer (**MIACP‐0**) showed no inhibitory activity (Figure 3b), confirming the charge‐dependent mechanism of inhibition.^[^ ^22^ ^]^

The similar viral inhibition activities of **MIP** (IC_50_ = 1.57 nM) and **MIACP‐1** (IC_50_ = 0.83 nM) confirm that ≈70% sulfation is optimal for maximum inhibition, aligning with previously reported degrees of sulfation required for maximal inhibitory activity. In fact, the relatively better inhibition observed for **MIACP‐1** compared to **MIP** is likely due to the presence of ≈30% carboxylate functionality, which may contribute additional inhibitory effects owing to its electronegative nature. A remarkably low half‐maximal inhibitory concentration (IC_50_ = 0.51 nM) highlights the excellent antiviral efficacy of these mucin‐inspired polymers against hRSV. Such low IC_50_ values make them among the most potent electronegative inhibitors reported for hRSV.**MIACP‐2** also exhibited a low IC_50_ value for hRSV‐GFP inhibition (IC_50_ = 1.82 nM), reinforcing their potential as effective antiviral agents.

The lower IC_50_ of **MIACP‐1** compared to **MIACP‐2** is likely due to the additional ≈30% carboxylate content in **MIACP‐1**. Commercially available porcine gastric mucin (PGM) and bovine submaxillary mucin (BSM) also showed hRSV‐GFP inhibition, but with significantly higher IC_50_ values (Figure 3c). This suggests that the synthetic inhibitors are more effective than commercially available natural mucins in terms of antiviral activity.

**MIACPs** exhibit strong charge‐dependent interactions with viral glycoproteins, as previously reported for polyglycerol sulfate groups, thereby inhibiting viral infection.^[^ ^13^ ^]^ Additionally, the long‐chain polysulfate amphiphilic elongated fibers of MIACPs likely engage in polyvalent interactions, providing sufficient steric shielding that contributes to their excellent inhibitory activity against hRSV. These features mark **MIACPs** as outstanding viral inhibitors.

**Figure 4:**
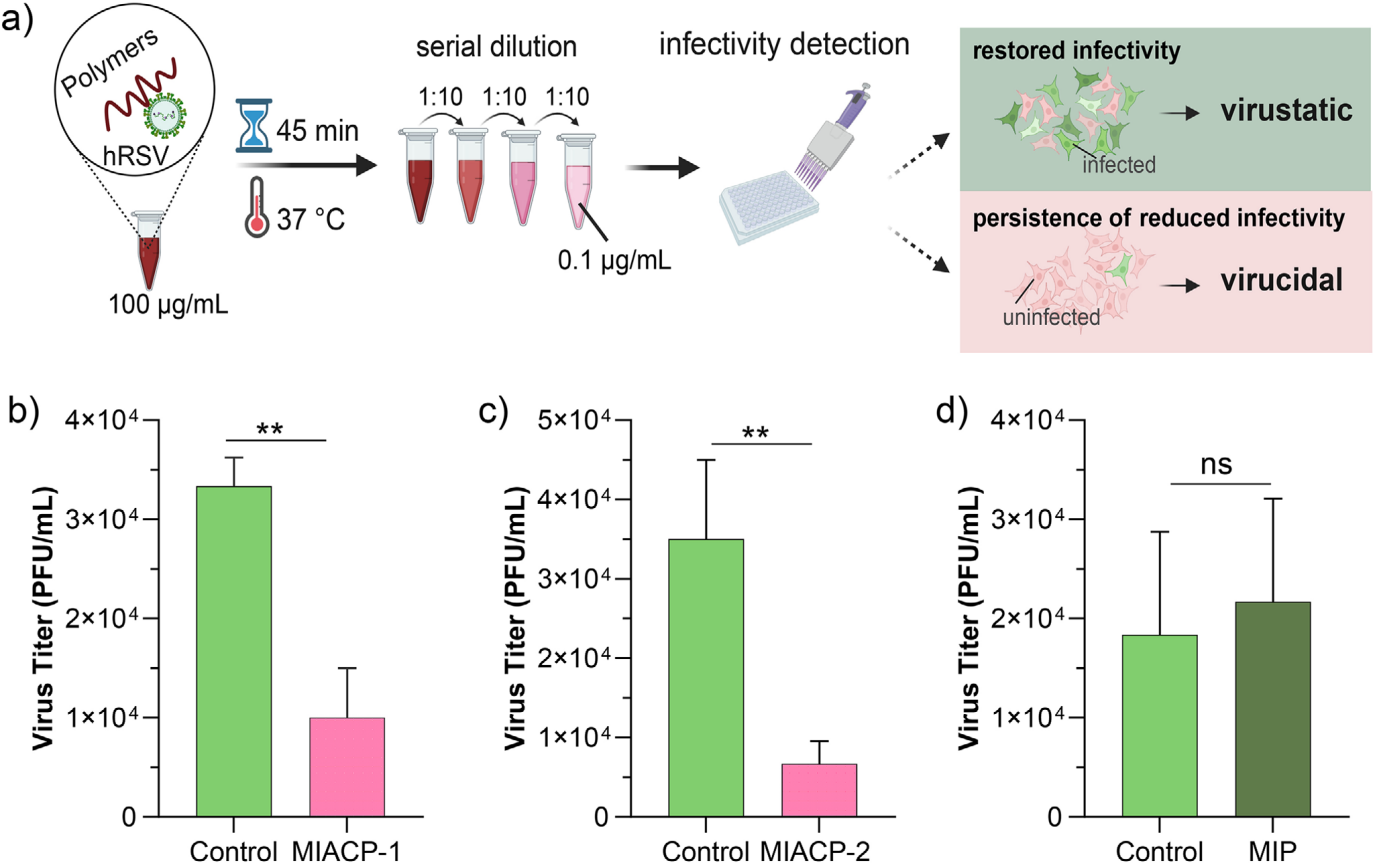
Serial dilution experiments for the evaluation of the virucidal efficacy of **MIACPs**: a) Schematic diagram of the workflow. Virucidal activity detection of b) **MIACP‐1**, c) **MIACP‐2** d) **MIP** against hRSV (*n* = 3). Viruses mixed with ddH_2_O were used as the control group. The error bars indicate mean ± SD. **p < 0.01; ns, *p *> 0.5.

We next wanted to investigate the virucidal activity of copolymers **MIACPs** through a dilution experiment.^[^ ^24^ ^]^ In this experiment, hRSV‐GFP (≈2 × 10^4^ PFU mL^−1^) was incubated with the desired amount of **MIACPs**, achieving a final concentration of 100 µg mL^−1^ (≈IC_90_ concentration), for 45 min at 37 °C. The virus‐**MIACPs** mixtures were then diluted 1:1000 fold into fresh medium, yielding a **MIACPs** concentration of 0.1 µg mL^−1^ (**Figure** **4**a). Then the diluted mixtures were applied to A549 cells to quantify the viral titer. At 24 h post‐infection, the viral titre was calculated by counting the number of infected cells per well. If the interaction between virus and **MIACPs** were reversible (virustatic), the dilution would be expected to promote the disassociation of **MIACPs** from the virus surface and restore the infectivity towards the control (≈90–100%, Figure 3b). Conversely, a virucidal activity would have prevented recovery after dilution. No notable recovery of viruses was observed after serial dilution in **MIACPs** treated groups, indicating that **MIACP‐1** and **MIACP‐2** are virucidal, confirming their strong binding to hRSV (Figure 4b,c). On the other hand, **MIP**, which lacks the C11 aliphatic group, did not exhibit any virucidal activity (Figure 4d). This further confirms the importance of the C11 hydrophobic functional groups in conferring virucidal efficacy.

### 2.5 Representation of the ECD Domain of hRSV and Its Possible Interaction with Inhibitors

Both the attachment protein G and the homotrimeric fusion protein F mediate viral binding to negatively charged heparan sulfate residues on the host cell surface,^[^ ^6^, ^50^ ^]^ providing a rationale for the inhibitory effect of the negatively charged polymer structures used in this study. It can be hypothesized that these structures interact with positively charged motifs of the ectodomain (ECD). It has been concluded that the binding of the G protein occurs via a stretch of positively charged amino acids (aa 187‐198: KRIPNKKPGKK; Uniprot P03423|GLYC_HRSVA) located between two mucin‐like motifs.^[^ ^7^, ^51^, ^52^ ^]^ To date, no motif has yet been identified for the ECD of the F protein. However, as we^[^ ^53^, ^54^ ^]^ and other groups^[^ ^55^, ^56^, ^57^ ^]^ have shown, analyzing the surface potential of the ECD of viral proteins can provide clues for binding sites. Nevertheless, this requires knowledge of the 3D structure. The ECD of the G protein essentially corresponds to an intrinsically disordered structure,^[^ ^58^ ^]^ as we have confirmed by an analysis using Alphafold3 (not shown). This makes it impossible to analyze the surface potential. However, it can be assumed that the flexibility of the structurally disordered ECD enables efficient interaction with negatively charged groups.

**Figure 5:**
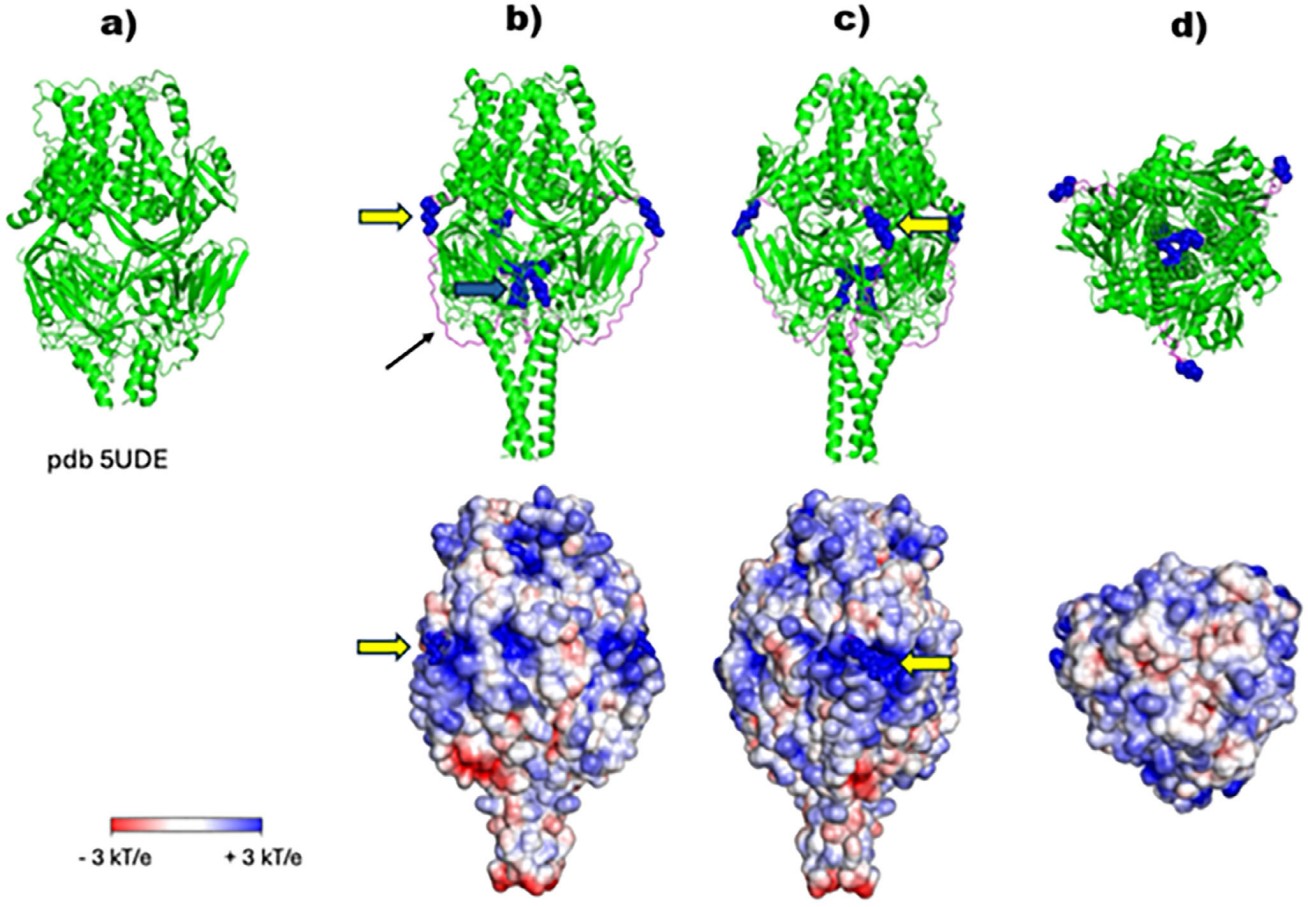
The surface potential of the ECD of human RSV. a) Crystal structure of the trimeric ECD of hRSV (pdb 5UDE). b–d) Upper part: 3D structure of the trimeric ECD of hRSV determined by Alphafold3. The sequence was taken from Uniprot P03423|GLYC_HRSVA. c) The structure depicted in (b) has been rotated by 90°. d) Top view. The sequence aa 75‐111, absent from the crystal structure (a), is shown in magenta (back thin arrow). Stretches of positively charged amino acids are shown as blue spheres (yellow arrow: RARR (aa 81‐84); blue arrow: KKRKRR (aa 106‐111)). Lower part: Surface potential of the ECD. The solvent accessible surface is shown. The calculation of the surface potential was performed using the ABPS plugin of PyMOL, with an ionic strength of 0.15 m and a pH of 7.0.

In contrast, the 3D structure of the ECD of the F protein has been experimentally elucidated (**Figure** **5**a). Recently, we have analyzed the surface potential of the crystal structure of the F ECD (PDB 5UDE).^[^ ^54^ ^]^ However, the latter is missing an essential domain (aa 75‐111) of the ECD which contains two stretches of positively charged amino acids. To take this domain into account, we modeled the 3D structure of the complete ECD sequence (Uniprot P03420|FUS_HRSVA) using Alphafold3. As illustrated in Figure 5b, the 3D structure aligns with the experimentally determined structure. The domain aa 75‐111 is depicted in magenta which could be described as intrinsically disordered structure. The positively charged amino acids of two stretches are shown as blue spheres. The sequence RARR (aa 81‐84) is exposed on the ECD surface, while the second sequence KKRKRR (aa 106‐111) is localized inside the trimer.

Figure 5d shows the surface potential of the ECD. It is evident that there are multiple regions of positive surface potential that have the potential to function as binding sites for negatively charged groups. Notably, the exposed stretch RRAR contributes to a distinct region of positive potential. This sequence exhibits a striking similarity to a Cardin‐Weintraub motif, which has been implicated as a binding motif for heparan sulfate residues.^[^ ^59^, ^60^ ^]^

## 3 Conclusion

In this work, using RAFT polymerization, we developed a series of high molecular weight amphiphilic copolymers (**MIACPs**) composed of ≈70% sulfated dendronized oligoglycerol methacrylate repeat units and ≈30% C11 aliphatic chain repeat units bearing terminal alkyl and carboxylate functionality. We also developed a homopolymer (**MIP**) of similar molecular weight, composed solely of sulfated dendronized methacrylate repeat units, without any C11 aliphatic functionality. The synthesized polymers were well characterized by ^1^H NMR, SEC, zeta potential, and elemental analysis. Cryo‐EM of the **MIACP**s confirmed a single‐chain filamentous structure. SANS corroborated the filamentous structure. The strategic statistical distribution of ≈30% aliphatic chains led to the formation of a single‐chain filamentous structure instead of an aggregated morphology.

**MIACPs** demonstrated good biocompatibility and less anticoagulant activity, as confirmed by cell viability assays across various cell lines and by anti‐complementary assays, respectively. A plaque‐reduction assay confirmed that **MIACPs** inhibit hRSV infectivity in a sulfate‐dependent manner, with activity significantly superior to commercially available natural mucins such as PGM and BSM. The excellent inhibitory activity is further supported by the possible interaction of the ECD domain of hRSV with the polymers. The inhibitory activity of **MIACPs** is comparable to that of **MIP**, confirming that ≈70% sulfation is optimal for effective virus inhibition. Additionally, **MIACPs** exhibited virucidal activity, as demonstrated by dilution experiments. In contrast, **MIP** did not exhibit any virucidity. Therefore, the strategy to develop **MIACPs** is unique in that they not only destroy the virus through their virucidal functionality, but also maintain strong inhibitory activity, despite having only 70% electronegative functionality. In addition, surface analysis of hRSV F revealed multiple potential **MIACPs** engagement sites, suggesting a high genetic barrier to resistance mutation, as single‐point substitutions are unlikely to abrogate inhibition. In addition, RSV F is a highly conserved protein, which makes it an ideal target for antiviral and vaccine‐based interventions.^[^ ^61^, ^62^ ^]^ These findings demonstrate that combining high sulfation and virucidal functionality within a filamentous polymer architecture is an effective strategy for developing potent antiviral materials.

## Conflict of Interest

The authors declare no conflict of interest.

## Supporting information



Supporting Information

Supporting Information

## Data Availability

The data that support the findings of this study are available in the supplementary material of this article.
