# Supplementary material for: Mucin‐Inspired Filamentous Sulfated Copolymers Effectively Inhibit Human Respiratory Syncytial Virus (hRSV) Infectivity
Source: Adv Sci (Weinh). 2025 Dec 1;13(9):e15908. doi: 10.1002/advs.202515908 (PMC12904038; doi:10.1002/advs.202515908)
Supplement: Supplementary file 1 — Supporting Information [file ADVS-13-e15908-s002.docx]

Supporting Information

**Mucin-Inspired Filamentous Sulfated Copolymers Effectively Inhibit Human Respiratory Syncytial Virus (hRSV) Infectivity**

Raju Bej,*^[a]^ Enyu Xie,^‡[c]^ Kai Ludwig,^[b]^ Robert F. Schmidt,^[d]^ Yannic Kerkhoff,^[b, f]^ Robert Dalgliesh,^[e]^ Nilanjan Paul,^[a]^ Michael Gradzielski, ^[d]^ Andreas Herrmann, ^[b]^ Christian Sieben* ^[c,g]^ and Rainer Haag*^[b]^

Dr. R. Bej, N. Paul

^[a]^Jyoti and Bhupat Mehta School of Health Sciences and Technology, Indian Institute of Technology Guwahati, 781039, Guwahati, India.

Email: rajubej@iitg.ac.in

Dr. K. Ludwig, Dr. Y. Kerkhoff, Dr. A. Herrmann, Dr. R. Haag

^[b]^Institut für Chemie und Biochemie, Freie Universität Berlin, Takustraße 3, 14195 Berlin, Germany.

Email: haag@zedat.fu-berlin.de

E. Xie, Dr. C. Sieben

^[c]^Nanoscale Infection Biology Group, Helmholtz Centre for Infection Research, Inhoffenstraße 7, 38124 Braunschweig, Germany.

Email: christian.sieben@helmholtz-hzi.de

Dr. R. F. Schmidt, Dr. M. Gradzielski

^[d]^ Stranski-Laboratorium für Physikalische und Theoretische Chemie, Institut für Chemie, Technische Universität Berlin, 10623 Berlin, Germany.

R. Dalgliesh

^[e]^ ISIS Pulsed Neutron and Muon Source, Science and Technology Facilities Council, Rutherford Appleton Laboratory, Harwell Oxford, Didcot OX11 0QX, U.K.

Dr. Y. Kerkhoff

^[f]^IT and Data Services, Zuse Institute Berlin, Takustraße 7, 14195 Berlin, Germany.

Dr. C.Sieben

^[g]^ Institute for Genetics, Technische Universität Braunschweig, Spielmannstr. 7, 38106 Braunschweig, Germany

‡ Contributed equally to this work

1. Experimental Section

**1.1 Materials and methods.**

All reagents and solvents were purchased from commercial suppliers and used without further purification. Benzoylated cellulose dialysis tubes (MWCO=2 kDa, 32 mm width), was purchased from Merck (Darmstadt, Germany). Bovine submaxillary mucin (BSM) was purchased from Merck and purified following the reported procedure before further use for experiments.^[1]^ Oligoglycerol based protected first generation dendrons (G1-OH) were synthesized following the literature procedure.^[2]^ Difunctional chain transfer agent (D) was synthesized following the literature procedure.^[3]^ Elemental composition determination was performed on a Vario EL CHNS element analyzer by Elementar Analysensysteme GmbH (Langenselbold, Germany). ^1^H NMR spectra were recorded on a Bruker AMX 500 MHz or 700 MHz (Bruker Corporation) or JEOL ECP 500 (JEOL GmbH) and the spectra were calibrated against TMS as the internal standard. Chemical shifts (δ) are reported in ppm via the deuterated solvent peak as the standard. Weight average molecular weight (MW) and polydispersity index (Ð) of the polymers were measured by size exclusion chromatography (SEC) using a Waters gel permeation chromatography (GPC) machine. GPC measurements in water were performed with an Agilent 1100 equipped with an automatic injector, isopump, and Agilent 1100 differential refractometer (Agilent Technologies, Santa Clara, CA, USA). The PSS Suprema (precolumn), 1× with pore size of 30 Å, 2× with pore size of 1000 Å (all of them with a particle size of 10 μm) column, was calibrated against Pullulan standards prior to measurements. Size exclusion chromatography (SEC) for THF soluble products was conducted on an SEC system (EcoSEC HLC8320GPC, Tosoh Bioscience, Japan) with a differential RI detector, polystyrene (Agilent EasiVial PSH 4.0 mL) as the standard, and THF as the eluent with a f low rate of 0.35 mL/min at 40 °C. The ζ-potential of the polymers were measured at 25 °C using a Zetasizer (Malvern Zetasizer-Nano ZS, Malvern Instruments Limited, Worcestershire, UK) and temperature equilibration for 60 s. Mass spectra were measured on a 6210 ESI-TOF and 6230 ESI-TOF from Agilent.

**1.2. Synthesis and characterization.**

**1.2.1 Monomer synthesis**: Monomers OGMA and NHSMA were synthesized using literature reported protocols. ^[4-5]^

**1.2.2 Synthesis of mucin-inspired amphiphilic copolymers (MIACPs):**

Synthesis of mucin-inspired copolymers (MIACPs) is depicted in Scheme S1.

**

**

**Scheme S1.** Synthesis of mucin-inspired amphiphilic copolymers (**MIACPs).**

**Synthesis of pOGMA-co-pNHSMA (P1)**

In a typical RAFT polymerization, the monomers OGMA (4.0 g, 10.3 mmol) and NHSMA (0.75 g, 4.12 mmol) were dissolved in 5.0 mL dry and degassed DMF in a glass ampule and to this the difunctional chain transfer agent (D) (12.4 mg, 0.021 mmol) was added. Here, the mol ratio of the monomer to chain transfer agent was kept ~690. After degassing for 5 min, required amount of AIBN (0.86 mg, 0.005 mmol) was added. Then the resulting mixture was transferred to a pre-heated oil bath at 75 °C and stirred at this temperature under argon atmosphere. The extent of polymerization was monitored by time dependent ^1^H NMR studies of the crude reaction mixtures and polymerization was stopped after 12 h when conversion of OGMA monomer was ~80 % and NHSMA monomer was around 75 %. Then the reaction was quenched by immersing the vessel inside liquid nitrogen and allowed to come back to room temperature. Then around 10.0 mL of dichloromethane (DCM) was added to it and the solution was transferred to dialysis tube (MWCO = 2 kDa) and purified by dialysis for 24 h by changing the outside DCM in regular interval. The dialyzed solution was dried under vacuum to obtain **pOGMA-co-pNHSMA (P1)** as slightly yellowish sticky liquid. In ^1^H NMR (**Figure S1**), the relative integration of the -CH protons from **pOGMA** (Ha) and –CH_2_ protons for NHS protons from **pNHSMA** (Hp) confirming incorporation of NHS units was ~ 30%. From the % of monomer conversion, the calculated degree of polymerization (DP) was 400 and 150 for **pOGMA** and **pNHSMA** unit respectively. The corresponding molecular weight of **P1** was estimated to be 183000 g/mol which matched with the SEC (**Figure S2**) determined molecular weight (*M_W_* = 128000 g/mol, *Ð* = 1.5) indicating well controlled RAFT polymerization. Yield: (70 %). ^1^H NMR (500 MHz, CDCl_3_) *δ* (ppm): 4.86 (bs, 1H), 4.23 (bs, 2H), 4.06 (bs, 2H), 3.85-3.51 (m, 10H), 2.88 (bs, 4H, -NHS protons), 1.94 (bs, polymer backbone, 2H), 1.4 (bs, 6H), 1.35 (bs, 6H),1.07-0.9 (bs, 3H).

**Substitution of the NHS groups by aliphatic C11 carbon chain amine (P2a and P2b)**

**P1** (1.2 g, 0.0065 mmol) and 11-amino undecanoic acid (235.5 mg, 1.17 mmol, 1.2 equivalents per NHS group) were dissolved in 10.0 mL dry DMF. Then required amount of triethyl amine (410.0 µL, 2.9 mmol, 3 equivalents per NHS group) was added and allowed the reaction to stir at 50 °C for 12 h under argon atmosphere. Then the reaction mixture was dialyzed against DCM for 24 h (MWCO = 2.0 kDa). The solution was collected, and the solvent was evaporated to get **P2a** as a semisolid. Yield: (90 %). Molecular weight: 185000 g/mol. ^1^H NMR (500 MHz, DMSO-d_6_) *δ* (ppm): 4.78 (bs, 1H), 4.16 (bs, 2H), 3.98 (bs, 2H), 3.62-3.42 (m, 12H), 1.84 (bs, polymer backbone, 2H), 1.35-0.85 (m, 31H).

**P2b** was synthesized using similar protocols using 11-amino undecane (200.4 mg, 1.17 mmol) as substituting material. Yield: (88 %). Molecular weight: 181000 g/mol. ^1^H NMR (500 MHz, DMSO-d_6_) *δ* (ppm): 4.78 (bs, 1H), 4.16 (bs, 2H), 3.98 (bs, 2H), 3.63-3.34 (m, 10H), 3.16 (bs, 2H), 1.84 (bs, polymer backbone, 2H), 1.35-0.85 (m, 36H).

**Deprotection of acetonide functional groups to synthesize P3a and P3b (MIACP-0)**

**P2a (**1.0 g, 0.0054 mmol) was dissolved in 14.0 mL of ethanol/water (10:4 v/v) mixture, and to this, required amount of aqueous HCl solution (37 %) was added to achieve the final concentration of reaction mixture 3.0 vol % HCl. The reaction mixture was stirred at room temperature for 6 h. After that, ethanol was evaporated, and the reaction mixture was dialyzed (MWCO = 2.0 kDa) against water for 24 h with regular replacement of the outside water. Finally, the solution was freeze-dried to obtain **P3a** as a white solid. In the ^1^H NMR (**Figure S5**) the peak corresponds to the acetonide group disappeared in **P3a** confirmed deprotection happened successfully. Yield: 85 %. Molecular weight: 153000 g/mol. ^1^H NMR (D_2_O, 500 MHz) *δ* (ppm): 4.96 (bs, 1H), 3.99-3.29 (m, 14H), 2.22-1.91 (bs, 4H), 1.4-0.91 (m, 19H).

**P3b (MIACP-0)** was synthesized using similar protocols. Molecular weight: 148000 g/mol. ^1^H NMR (D_2_O, 500 MHz) *δ* (ppm): 4.97 (bs, 1H), 3.87-3.56 (m, 14H), 2.97 (bs, 2H), 1.94 (bs, polymer backbone, 2H), 1.42-0.85 (m, 24H).

**Sulfation of copolymer to synthesize MIACPs**

Sulfation of the polymers was performed as follows: Firstly, **P3a** (0.8 g, 0.0052 mmol) was dissolved in 20.0 mL dry DMF, then solution of sulphamic acid (NH_3_SO_3_) (2.02 g, 20.8 mmol, 2.5 equivalent per hydroxyl group to be sulfated, total number of hydroxyl groups = 1600) was added and allowed the reaction to stir at room temperature for 72 h. Then the reaction was quenched by addition of 20.0 mL water. The pH of the reaction mixture was adjusted by dropwise addition of saturated sodium hydrogen carbonate solution until pH 7.4. The solution was transferred to dialysis tube and dialyzed against saturated solution of sodium chloride (NaCl) for 24 h, then concentration of NaCl was gradually decreased to distilled water for 24 h and finally against distilled water for 24 h. The collected solution was freeze dried to obtained **MICP-1** as white solid material. The degree of sulfation was quantified from elemental analysis. In the ^1^H NMR spectrum, the protons adjacent to the sulfate groups shifted to down field region confirming addition of electronegative sulfate groups. Yield: (80 %). ^1^H NMR (D_2_O, 700 MHz) *δ* (ppm): 5.01 (bs, 1H), 4.38-4.27 (m, 6H), 3.86 (bs, 8H), 3.02 (bs, 2H), 2.09 (bs, 4H), 1.33-0.99 (m, 19H). ^1^H determined molecular weight considering 550 repeat units and 94 % degree of sulfation = 320 kDa. SEC: Molecular weight (*M_W_)* = 290000 g/mol.

Similar protocol was followed for the sulfation of **P3b (MIACP-0)** to prepare **MIACP-2.** Yield: (82 %). ^1^H NMR (D_2_O, 700 MHz) *δ* (ppm): 5.01 (bs, 1H), 4.38-4.27 (m, 6H), 3.86 (bs, 8H), 3.01 (bs, 2H), 2.11 (bs, polymer backbone, 2H), 1.37-0.96 (m, 24H). ^1^H determined molecular weight considering 550 repeat units and 92 % degree of sulfation = 310 kDa. SEC: Molecular weight (*M_W_)* = 285000 g/mol.

**1.2.3 Synthesis of mucin-inspired polymers (MIP):**

Synthesis of mucin-inspired polymer (MIP) is depicted in Scheme S2.

**

**

**Scheme S2.** Synthesis of mucin-inspired polymer (**MIP)**

**Synthesis of POGMA (P4)**

In a typical RAFT polymerization, the monomer OGMA (4.0 g, 10.3 mmol) was dissolved in 4.0 mL dry and degassed DMF in a glass ampule and to this the difunctional chain transfer agent (D) (10.3 mg, 0.017 mmol) was added. Here, the mol ratio of monomer to chain transfer agent was kept ~ 605. After degassing for 5 min, required amount of AIBN (0.7 mg, 0.0043 mmol) was added. Then the resulting mixture was transferred to a pre-heated oil bath at 75 °C and stirred at this temperature under argon atmosphere. After 12 h of polymerization, the reaction was quenched by immersing the vessel inside liquid nitrogen and allowed to come back to room temperature. Then around 6.0 mL of dichloromethane (DCM) was added to it and the solution was transferred to dialysis tube (MCWO = 2 kDa) and purified by dialysis for 24 h by changing the outside DCM in regular interval. The dialyzed solution was dried under vacuum to obtain P4 as slightly yellowish sticky liquid. The crude ^1^H NMR analysis suggested ~ 83 % monomer conversion. From the % of monomer conversion, the degree of polymerization (DP) was calculated to be 500 and corresponding molecular weight of **P4** was estimated to be 195000 g/mol which matched with the SEC (**Figure S12**) determined molecular weight (*M*_w_ = 180000 g/mol, *Ð* = 1.5) indicating well controlled RAFT polymerization. Yield: (78 %). ^1^H NMR (CDCl_3_, 500 MHz) *δ* (ppm): 4.83 (bs, 1H), 4.22 (bs, 2H), 4.05 (bs, 2H), 3.85-3.45 (m, 10H), 1.84 (bs, polymer backbone, 2H), 1.4 (bs, 6H), 1.34 (bs, 6H),1.02-0.89 (bs, 3H).

**Deprotection of acetonide functional groups**:

**P4** (3.0 g, 0.008 mol) was dissolved in 42.0 mL of ethanol/water (10:4 v/v) mixture, and to this, required amount of aqueous HCl solution (37%) was added to achieve the final concentration of reaction mixture 3.0 vol % HCl. The reaction mixture was stirred at room temperature for 6 h. After that, ethanol was evaporated, and the reaction mixture was dialyzed (MCWO = 2.0 kDa) against water for 24 h with regular replacement of the outside water. Finally, the solution was freeze-dried to obtain **P5** as a white solid. In the ^1^H NMR the peak at 1.4 ppm and 1.34 ppm corresponds to the acetonide group disappeared in **P4** confirmed deprotection happened successfully. Yield: 90 %. ^1^H NMR (D_2_O, 500 MHz) *δ* (ppm): 4.97 (bs, 1H), 3.88-3.57 (m, 14H), 1.94 (bs, polymer backbone, 2H), 1.10-0.97 (m, 3H).

**Sulfation of polymer to synthesize MIP**

Sulfation of the polymers was performed as follows: Firstly, **P5** (2.3 g, 7.46 mmol, based on the molecular weight of repeat unit to be sulfated) was dissolved in 20.0 mL dry DMF, then solution of sulphamic acid (NH_3_SO_3_) (7.24 g, 74.6 mmol, 2.5 equivalent per hydroxyl group to be sulfated) was added and allowed the reaction to stir at room temperature for 72 h. Then the reaction was quenched by addition of 20.0 mL water. The pH of the reaction mixture was adjusted by dropwise addition of saturated sodium hydrogen carbonate solution until pH 7.4. The solution was transferred to dialysis tube and dialyzed against saturated solution of sodium chloride (NaCl) for 24 h, then concentration of NaCl was gradually decreased to distilled water for 24 h and finally against distilled water for 24 h. The collected solution was freeze dried to obtained MIP as white solid material. The degree of sulfation was quantified from elemental analysis. In the ^1^H NMR spectrum (**Figure S14**), the protons adjacent to the sulfate groups shifted to down field region confirming addition of electronegative sulfate groups. Yield: (78 %). ^1^H NMR (D_2_O, 700 MHz) *δ* (ppm): 4.94 (bs, 1H), 4.32-4.21 (m, 6H), 3.8-3.65 (m, 8H), 1.97 (bs, polymer backbone, 2H), 1.28-1.0 (m, 3H). ^1^H determined molecular weight considering 500 repeat units and 94 % degree of sulfation = 350 kDa. SEC: Molecular weight (*M_W_)* = 312000 g/mol.

**1.3. Solution preparation.**

A stock polymeric solution (1.0 mg/mL) was prepared in Milli-Q water by directly dissolving a given polymer in measured amount of water. This stock solution was directly used for Cryo-EM and zeta potential measurement. Another stock polymeric solution (10.0 mg/mL) was prepared in Milli-Q water. This stock solution was directly used for biocompatibility assay and virus experiments.

**1.4. Cryo-electron Microscopy and Tomography.**

A perforated (1 µm hole diameter) carbon film-covered microscopical 200 mesh grids (R1/4 batch of Quantifoil, MicroTools GmbH, Jena, Germany) was cleaned with chloroform and hydrophilized by 60 s glow discharging at 8 W in a BALTEC MED 020 device (Leica Microsystems, Wetzlar, Germany). Then 4.0 µL of the corresponding polymer solution was applied to the hydrophilized grid. The sample was vitrified by automatic blotting and plunge freezing with a FEI Vitrobot Mark IV (Thermo Fisher Scientific Inc., Waltham, Massachusetts, USA) using liquid ethane as cryogen. The vitrified grid was stabilized by a copper auto grid and fixed by a spring clamp under liquid nitrogen. The thus prepared auto grids were transferred under liquid nitrogen into a Talos Arctica transmission electron microscope (ThermoFisher Scientific Inc., Waltham (MA), USA) using the microscope's autoloader transfer routine. The microscope operated at an accelerating voltage of 200 kV, and images were acquired with a FEI Falcon 3 direct electron detector (Thermo Fisher Scientific Inc., Waltham, Massachusetts, USA) using the microscope's low-dose protocol.

Tomograms were acquired with the same microscope using a Volta phase plate. Single axis tilt series (±64° in 2° tilt angle increments) were performed using the FEI Tomography software (Version 4.5.0, ThermoFisher Scientific Inc., Waltham (MA), USA) recorded with a Falcon 3 direct electron detector at full resolution (28 K primary magnification) with a total dose less than 120 e-/Å2. Tomogram reconstruction was done using ThermoFisher Inspect3D software with binning factor 2. For semi-automatic analysis the cyro-ET images were pre-processed in Fiji with contrast enhancement, and 3D Gaussian and 3D median filter with subsequent Triangle thresholding. Of the binarized images only particles with an area of more than 50 pixels were retained and skeletonized. The resulting binary skeleton of the amphiphilic elongated fibre structures was analyzed with the Analyze Skeleton (2D/3D) plugin. The longest shortest paths of the resulting structures were regarded as the fibre backbones and used to determine the fibre length distribution. The distribution showed three distinct populations. Objects shorter than 10 nm are clearly noise and objects smaller 75 nm were regarded as fibre fragments. Longer structures were regarded as unfragmented fibres which was confirmed by manual tracing and evaluation.

**1.5. Small Angle Neutron Scattering (SANS)**

**Experimental**

The small-angle neutron scattering (SANS) experiments were performed on the Larmor instrument at ISIS Pulsed Neutron and Muon Source (Didcot, United Kingdom; experiment RB2220343 ^[6]^) using a temperature-controlled sample changer and rectangular quartz cuvettes of 2 mm thickness. The temperature was fixed at 25 °C. Neutron wavelengths $\lambda$ of 0.9 to 13 Å were used simultaneously by time-of-flight, yielding a total $q$-range of 4.5 x 10^-3^ to 6.7 x 10^‑1^ Å^‑1^. Here, $q=\left( {4\pi}/\lambda\right)\sin(\theta/2)$ is the magnitude of the scattering vector, where $\theta$ is the scattering angle. Data reduction was done using the MANTID software.^[7]^ The raw intensity data were corrected for background scattering and weighted by the transmission of the sample.^[8]^ The absolute scaling was performed using a secondary calibrated polymer blend sample.^[9]^ The incoherent background scattering was subtracted by fitting the Porod law:

$I\left( q \right)=\frac{A}{q^{n}}+\mathrm{bkg}$ (1)

to the experimental data for *q* > 0.1 Å^-1^. Here, A and n are constants and bkg is the incoherent background scattering intensity.

**1.6. Cell viability assay.**

The cytotoxicity of the polymers was analyzed by the cell viability assay Cell Counting Kit 8 (CCK-8) from Sigma-Aldrich Chemie GmbH (Taufkirchen, Germany) according to the manufactures’ instructions. For the assays, three cell lines were used: A549 human lung carcinoma cells (DSMZ ACC 107), 16HBE14o- human bronchial epithelial cells (Millipore SCC150) and Vero E6 African green monkey kidney epithelial cells (ATCC CRL-1586). Cells were cultured in Dulbecco’s Modified Eagle Medium (DMEM) supplemented with 10% fetal bovine serum (FBS), penicillin/streptomycin and GlutaMAX (all from Gibco BRL, Eggenstein, Germany). The cells were passaged every 3 to 4 days after reaching 70 % to 90 % confluency. For the assay, cells were seeded in a 96 well plate (4.000 cells/well) and incubated overnight at 37 °C and 5% CO_2_. Then, compounds were added in serial dilutions in triplicates. SDS (1%) and non-treated cells served as a control. For background subtraction, wells without cells were used. Cells were incubated for another day at 37 °C before CCK-8 solution was added. After approximately 3 hours absorbance was measured at a measurement wavelength of 450 nm and a reference wavelength of 650 nm with a plate reader (Infinite ® M200 PRO, Tecan Group Ltd., Männedorf, Switzerland). The assay was repeated three times. The cell viability was calculated by setting the non-treated control with cells to 100% after subtracting the background.

**1.7. Anticoagulant activity.**

Anticoagulant activity was determined by measuring the activated partial thromboplastin time (aPTT) of standard plasma mixed with different concentrations of the test compounds. For this, 50.0 µL standard plasma (Siemens Healthcare #ORKL17) was combined with 50.0 µL Actin FS (Siemens Healthcare #B4218-20) and 2.0 µL of the test compounds dilutions. The mixture was incubated for 3 minutes at 37°C, then the reaction was started by adding 50.0 µL of CaCl2 solution (Siemens Healthcare #ORH037) and the time until clot formation was recorded on a coagulometer (STart Max, Stago).

**1.8. Virus inhibition studies.**

A549 cells were seeded in a 96-well plate with a cell density of 2×10^4^ cells per well and incubated overnight at 37 °C with 5% CO_2_. RSV-GFP (human respiratory syncytial virus subtype A long strain with a GFP reporter gene) were pre-incubated with desired concentration of compounds for 45 min at 37 °C, and then added on cells. 2 hours later, the inoculum was removed and replaced with infection medium (2% FBS in DMEM). Cells were then cultured at 37 °C with 5% CO_2._ At 24 hours post infection, cells were imaged, and the number of infected cells was detected through Incucyte (IncuCyte S3, Sartorius, Germany). Viruses incubated with double-distilled water (ddH_2_O, the solvent of the compounds) were used as control. The results were exhibited with relative inhibition ratios that were calculated by comparing numbers of infected cells of compound treatment group to that of control group. Statistical analysis to generate IC_50_ values was conducted using GraphPad Prism software (version 9.4.1).

**1.9. Virucidal assays.**

A549 cells were seeded in a 96-well plate with a cell density of 2 × 10^4^ cells/well and incubated overnight at 37 °C with 5% CO_2_. RSV-GFP (~2×10^4^ PFU) was incubated with 100  μg/mL of desired polymers for 45 min at 37 °C. A control group was created by mixing RSV-GFP with ddH_2_O. Then the virus-compounds mixture was subjected to three sequential 10-fold dilutions in fresh infection medium to reach a 1:1000 fold dilution. The final concentration of polymers in the highest diluted mixture is 0.1 µg/mL. 50 μL of the 1:1000 fold dilution mixture was then added to cells and incubated for 2 hours. The inoculum was removed and replaced with infection medium (2% FBS in DMEM). Cells were then cultured at 37 °C with 5% CO_2_. At 24 hours post infection, cells were imaged, and the number of infected cells was detected through Incucyte. The virus titer was calculated through the number of infected cells per well.

**1.10. Methods: Surface Potential of the ECD of human RSV**

The three-dimensional structure of the full-length F protein of human RSV was generated using the AlphaFold3 algorithm^1^(https://alphafoldserver.com). The amino acid sequence was obtained from the UniProt database (see https://www.uniprot.org/uniprotkb/P09429/entry).

3D structures were visualized using PyMOL (license information: PyMOL Invoice #51548). The surface potential was calculated on the APBS server (Advanced-Poisson-Boltzmann) using the corresponding plugin in PyMOL.

**2.Additional Figures**

**
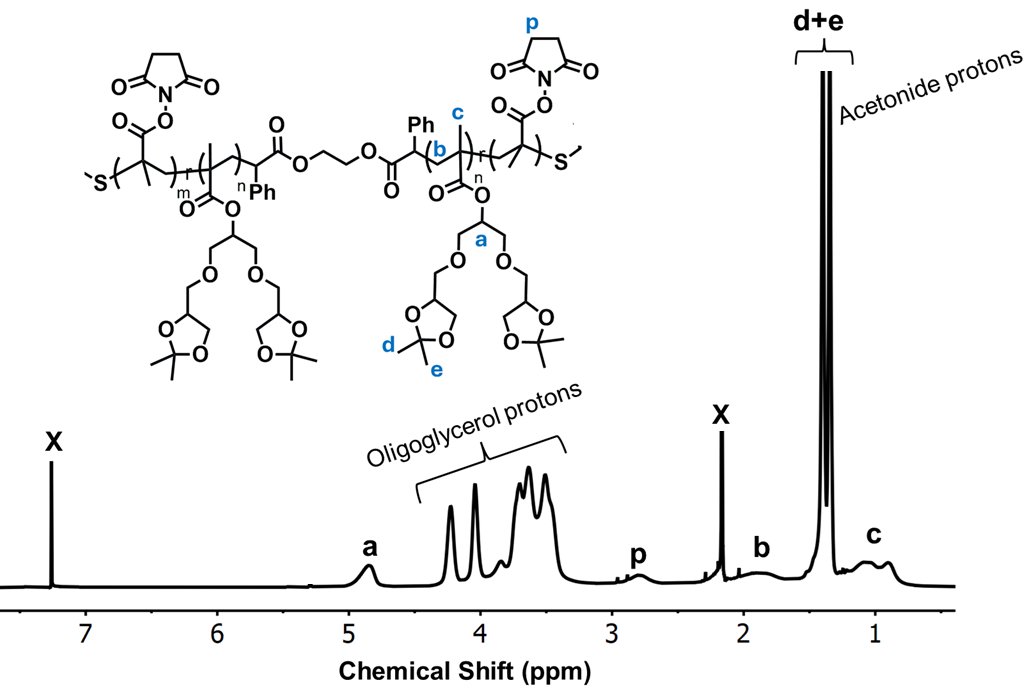
**

***Figure S1****. ^1^H NMR spectrum of* ***pOGMA-co-pNHSMA (P1)*** *in CDCl_3_. X indicates peaks from residual solvent.*


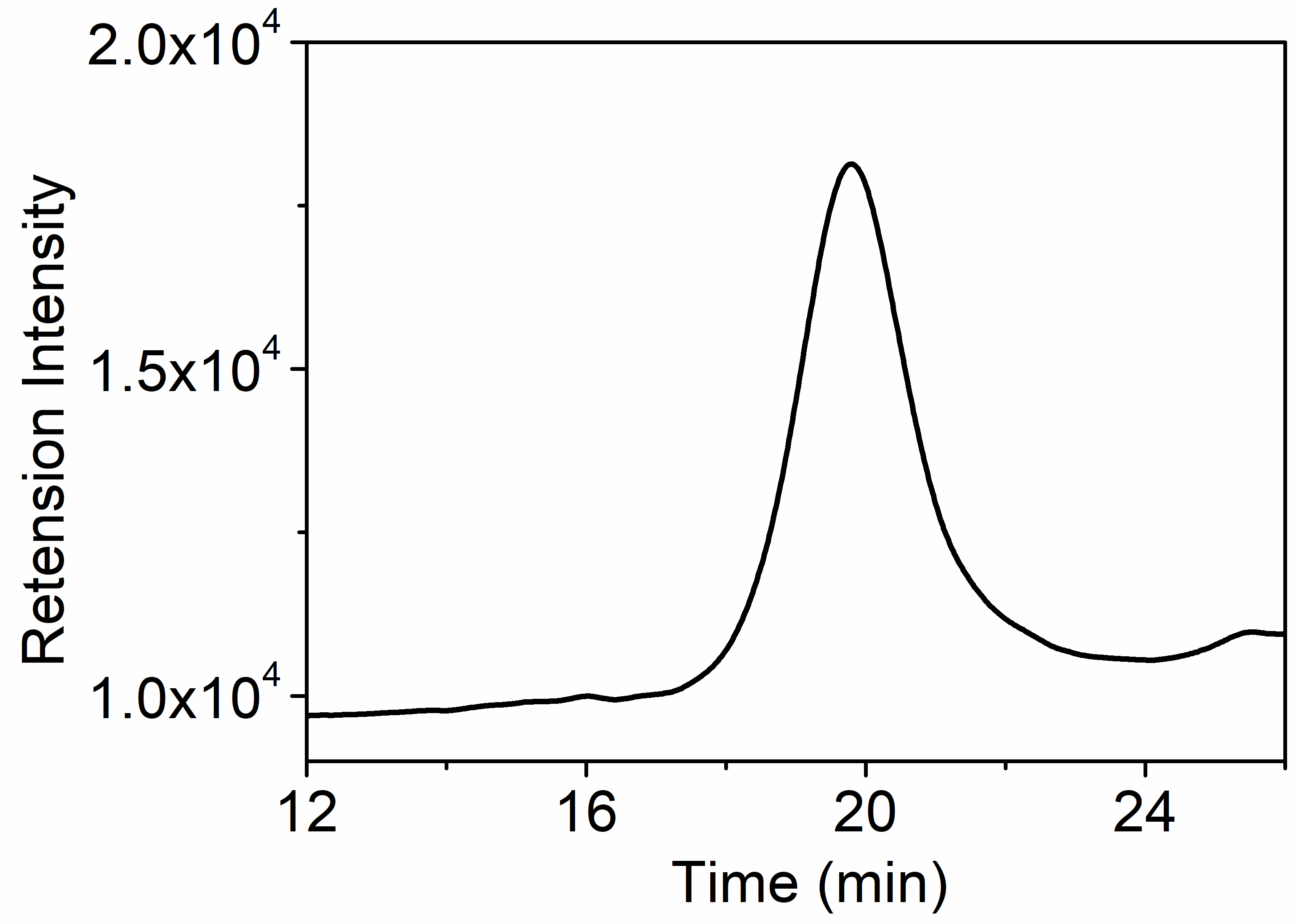


***Figure S2****. SEC trace of* ***POGMA-co-PNHSMA*** *in THF.*

***
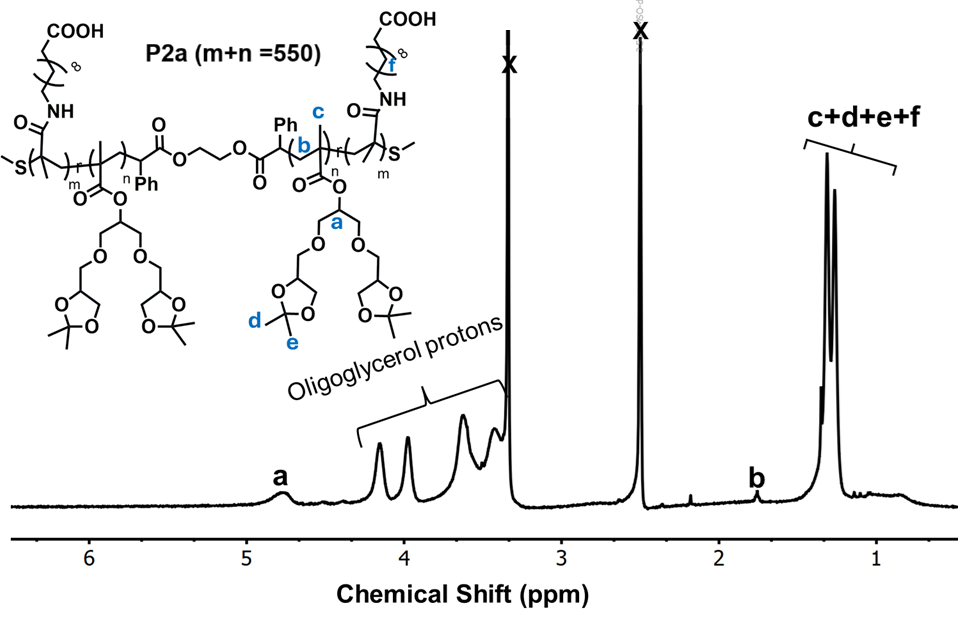
Figure S3****. ^1^H NMR spectrum of* ***P2a*** *in DMSO-d_6_. X indicates peaks from residual solvent.*

***
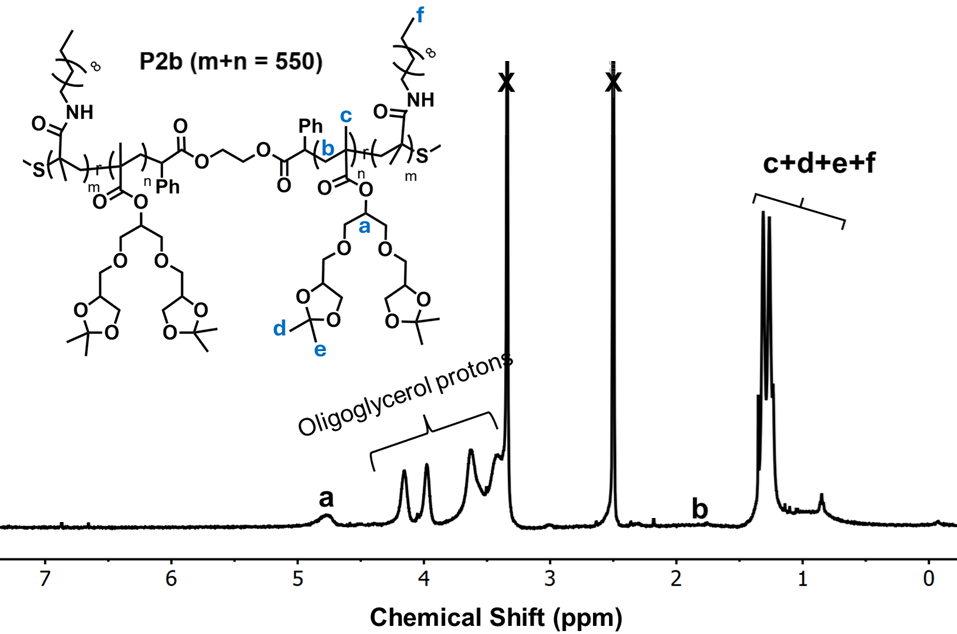
Figure S4****. ^1^H NMR spectrum of* ***P2b*** *in DMSO-d_6_. X indicates peaks from residual solvent.*

***
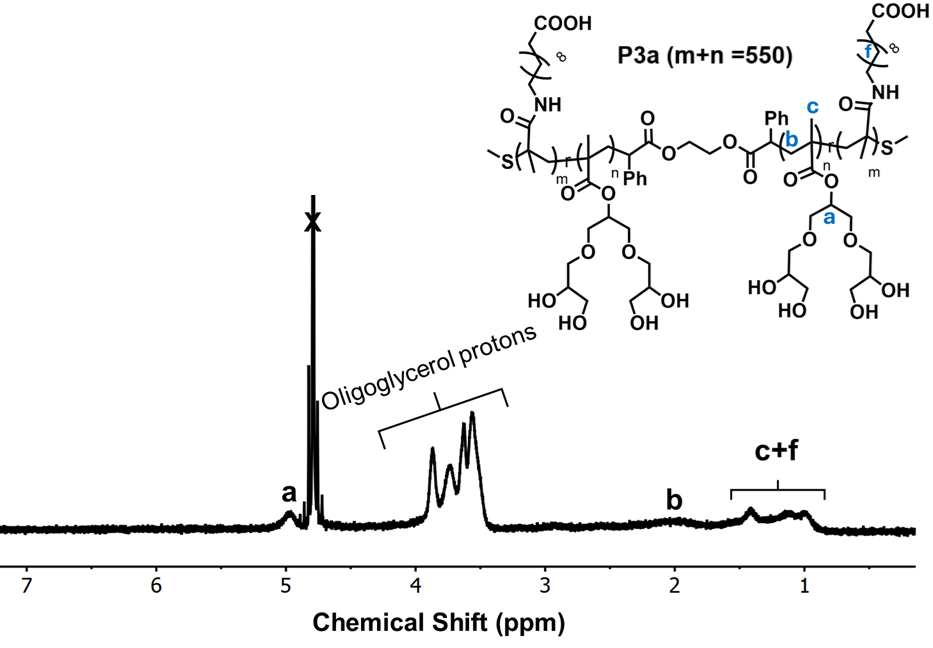
Figure S5****. ^1^H NMR spectrum of* ***P3a*** *in D_2_O. X indicates peaks from residual solvent.*

***
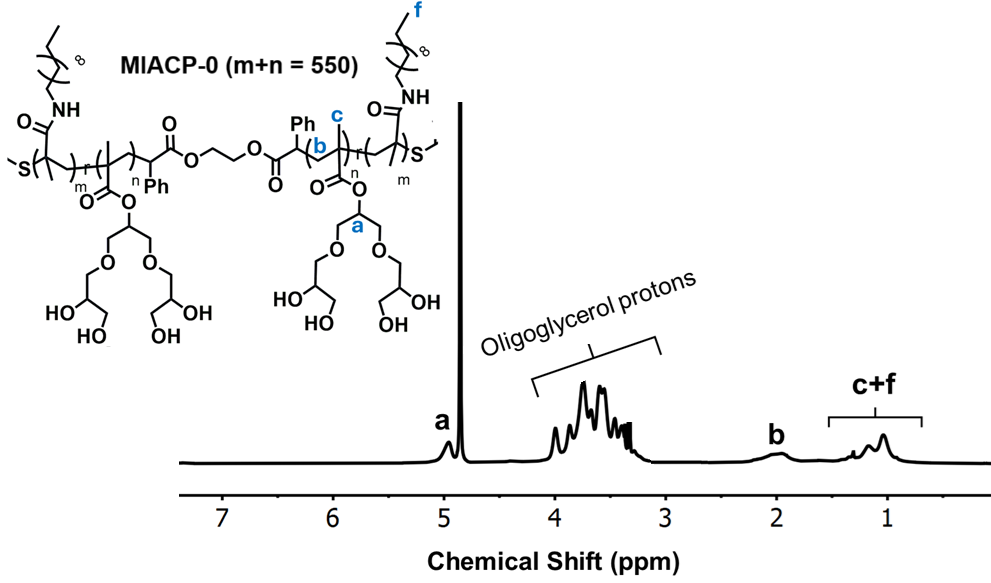
Figure S6****. ^1^H NMR spectrum of* ***P3b (MIACP-0)*** *in D_2_O. X indicates peaks from residual solvent.*

***
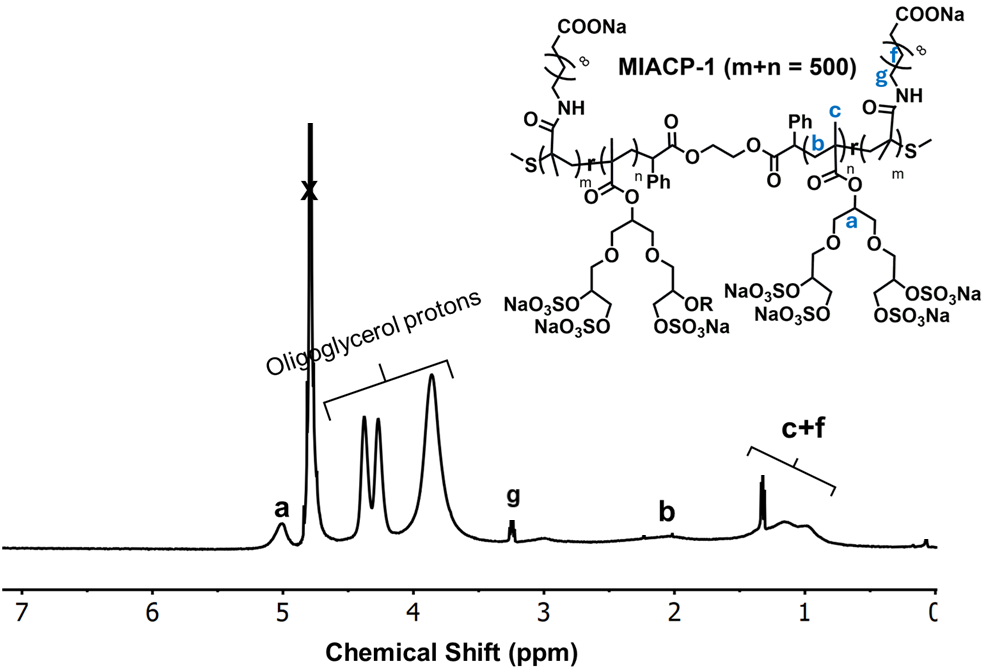
Figure S7****. ^1^H NMR spectrum of* ***MIACP-1*** *in D_2_O. X indicates peaks from residual solvent.*

*
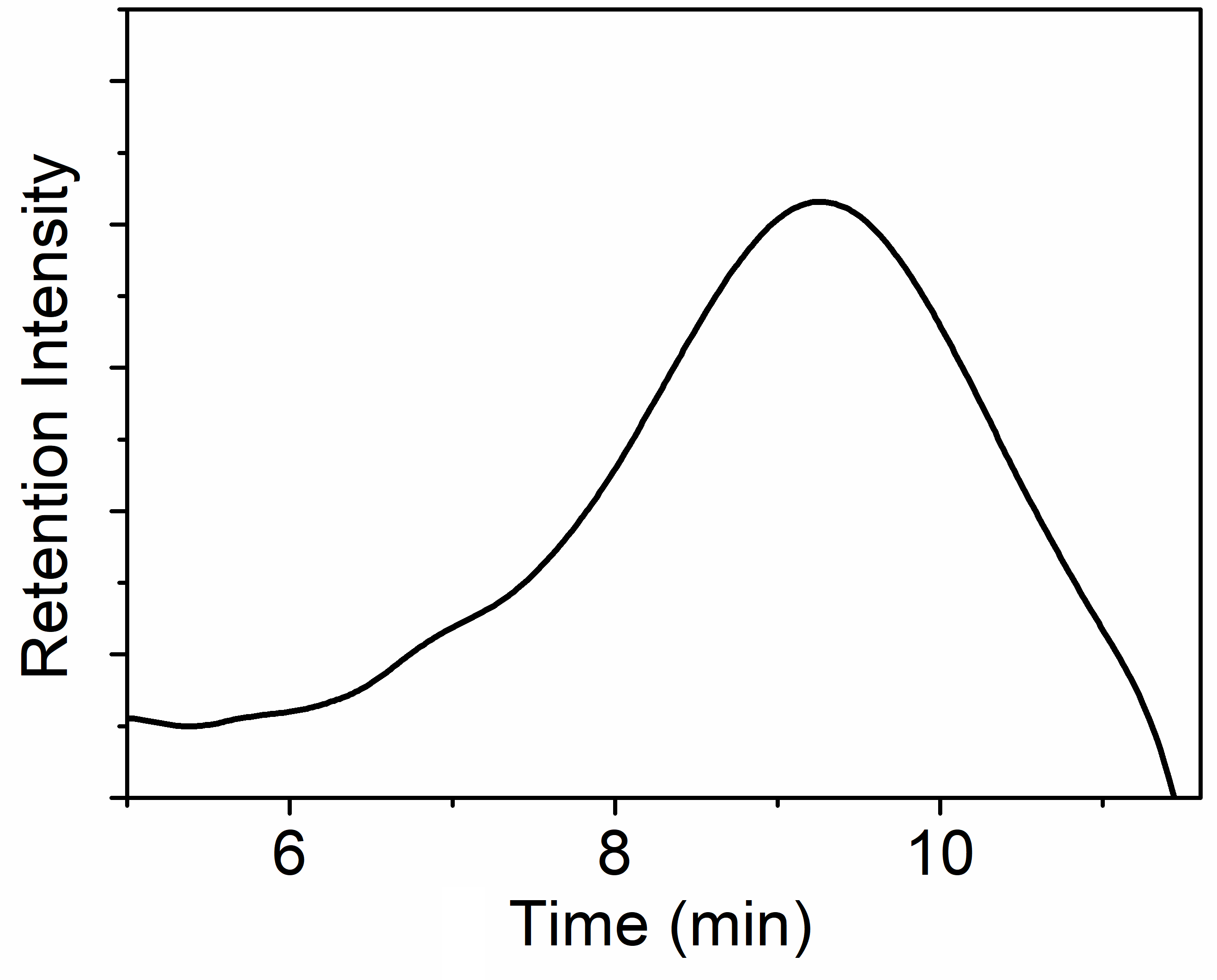
*

***Figure S8****.SEC traces of* ***MIACP-1*** *in water.*

***
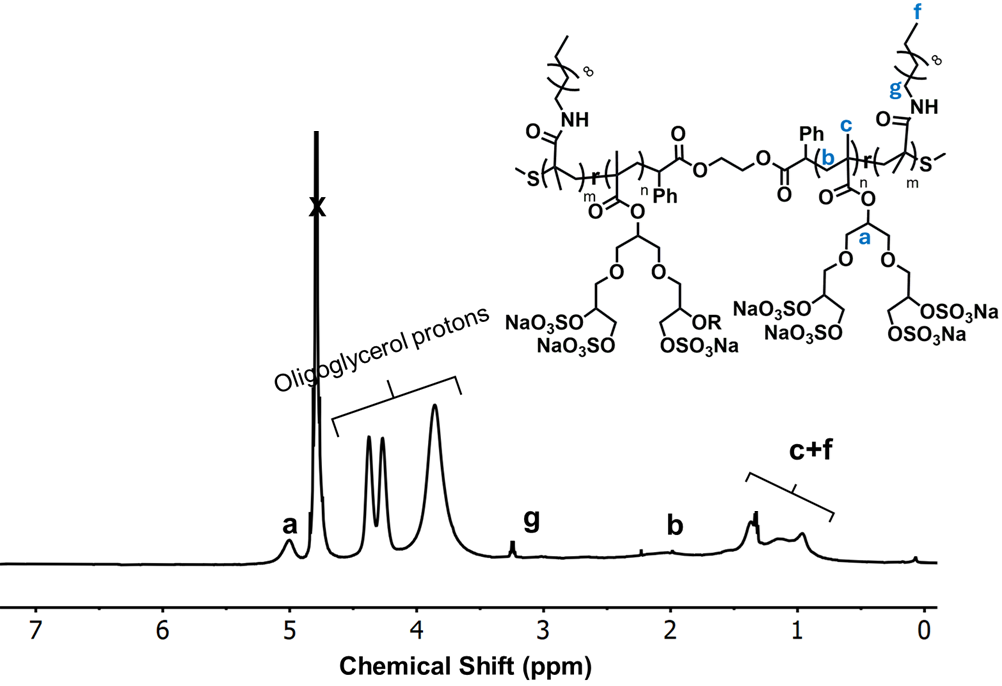
Figure S9****. ^1^H NMR spectrum of* ***MIACP-2*** *in D_2_O. X indicates peaks from residual solvent.*

*
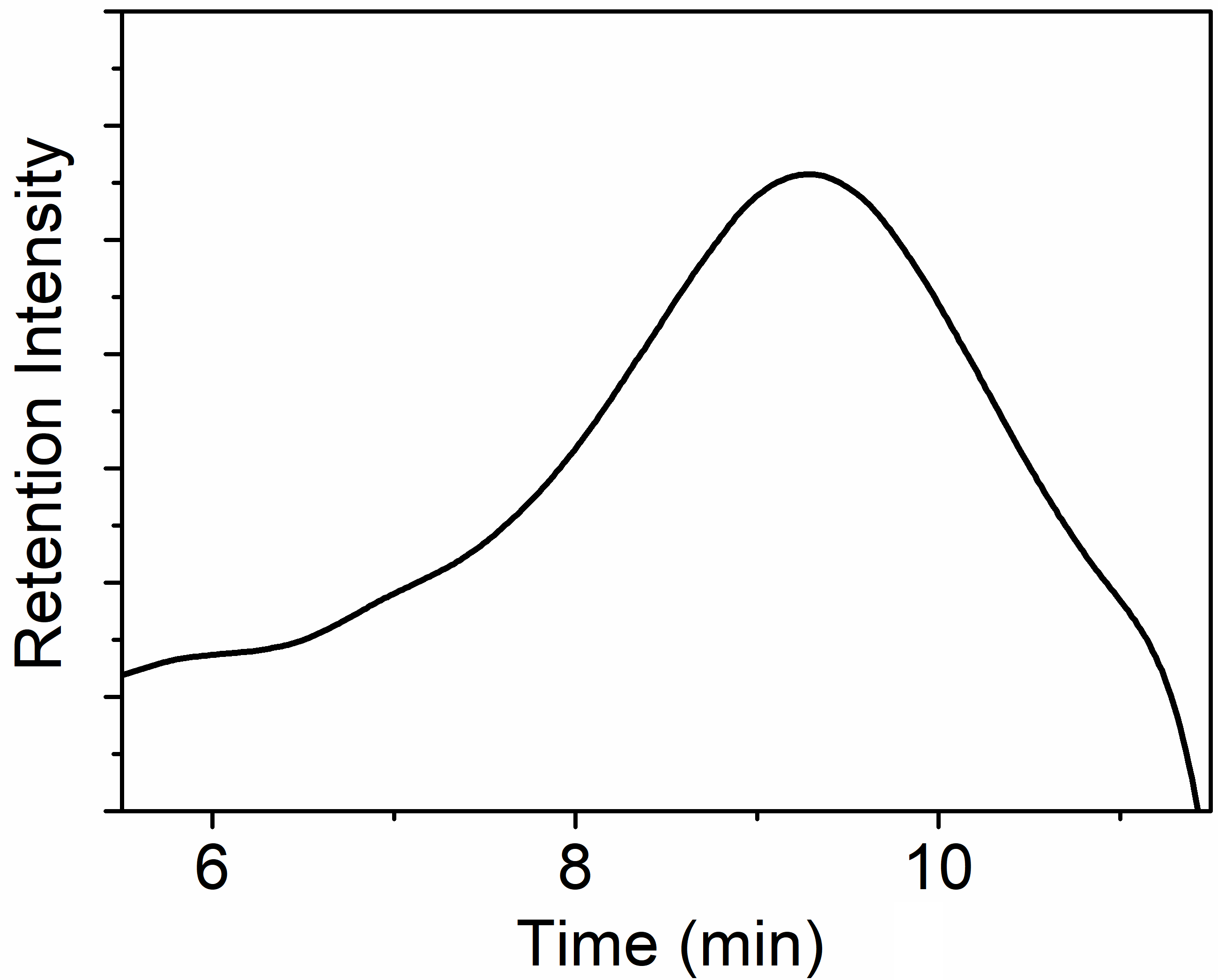
*

***Figure S10****. SEC traces of* ***MIACP-2*** *in water.*

*.*

***
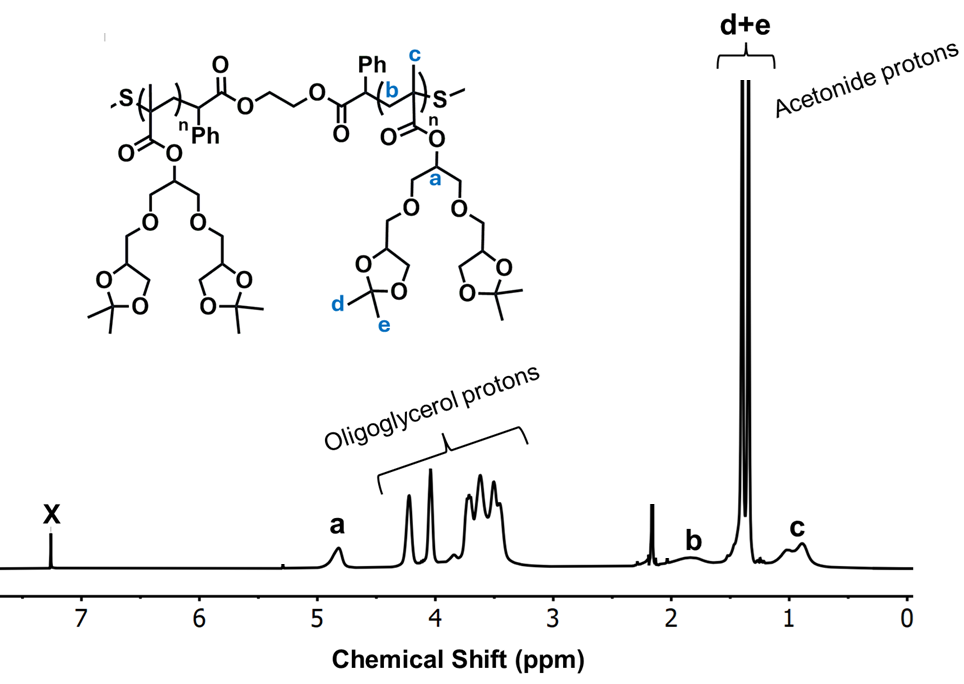
Figure S11****. ^1^H NMR spectrum of* ***pOGMA (P4)*** *in CDCl_3_. X indicates peaks from residual solvent.*


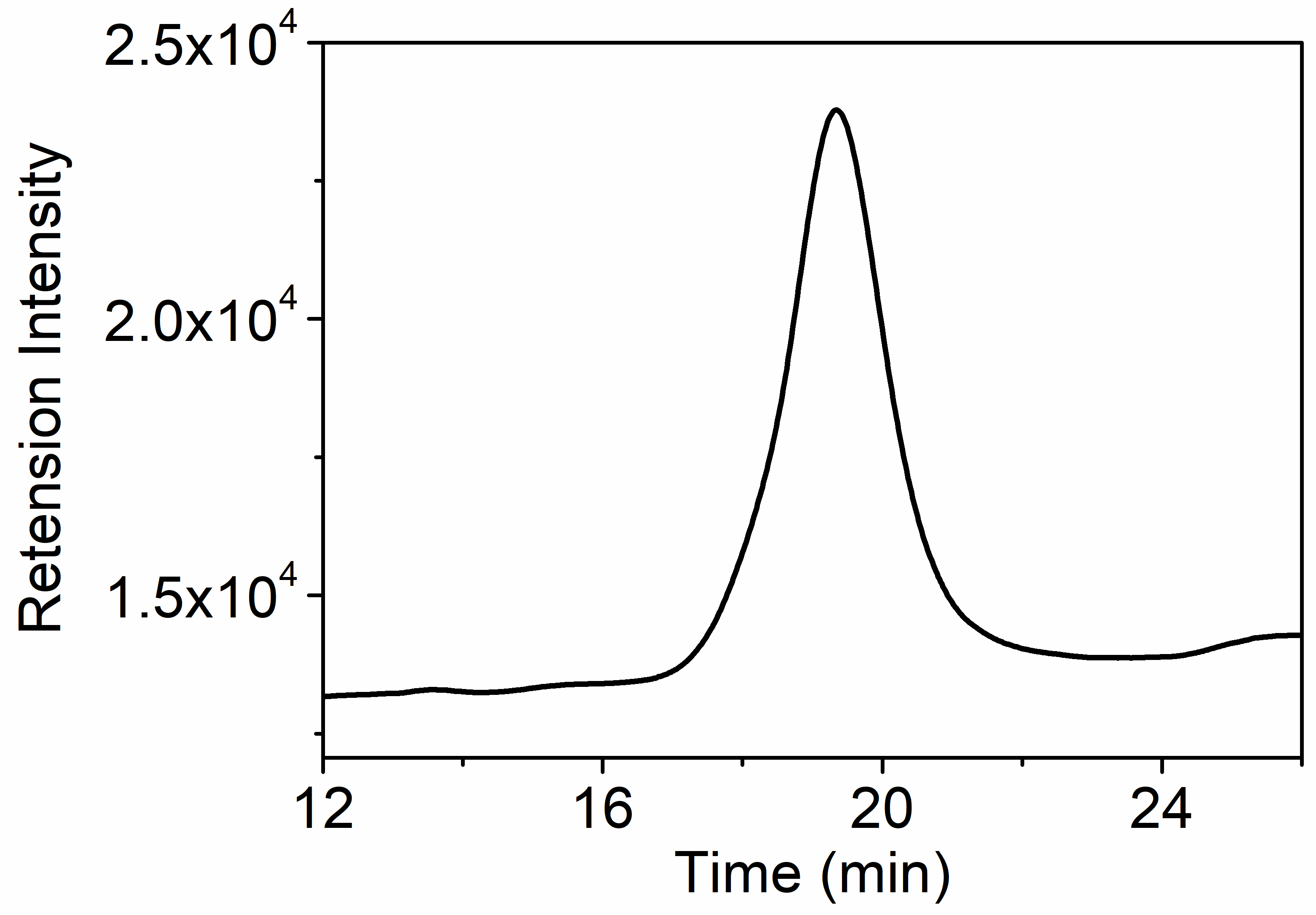


***Figure S12****. SEC trace of* ***pOGMA (P4)*** *in THF.*

***
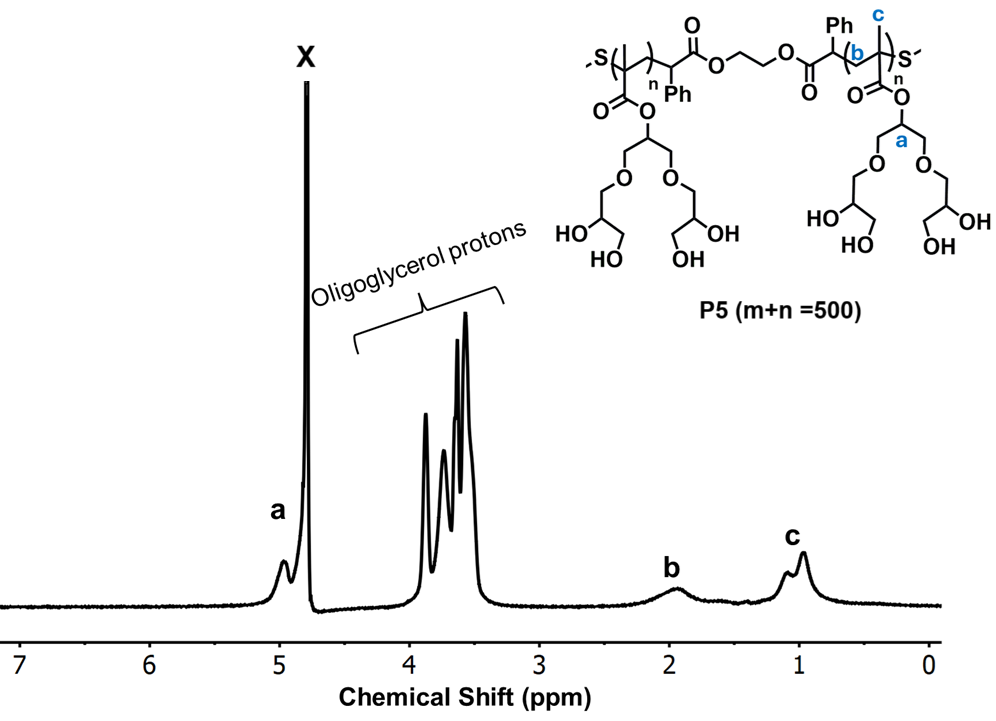
Figure S13****. ^1^H NMR spectrum of* ***P5*** *in D_2_O. X indicates peaks from residual solvent.*

***
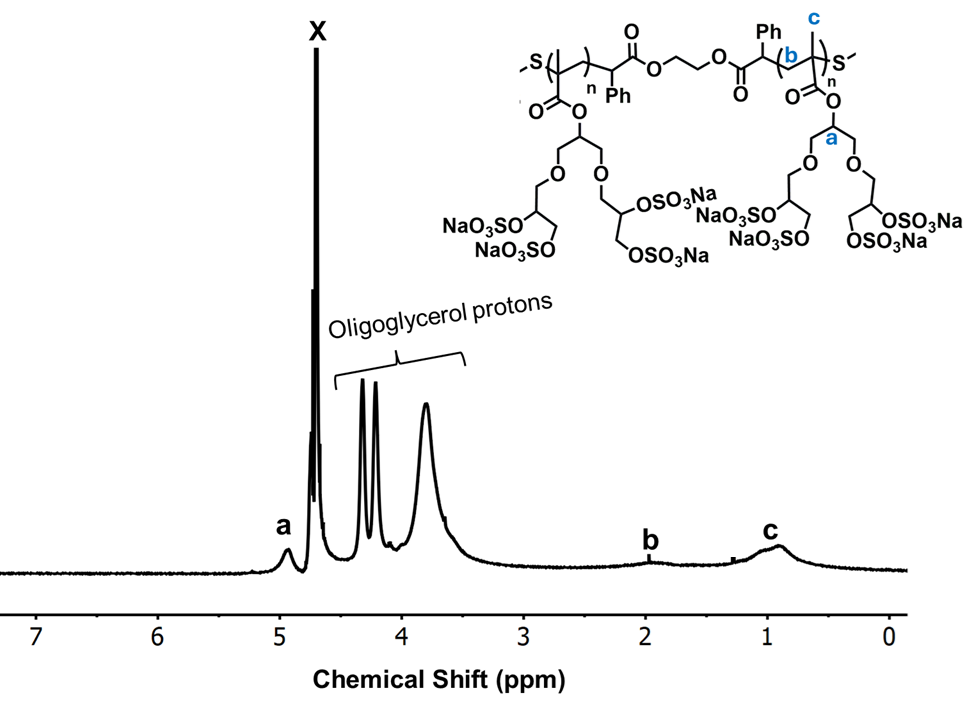
Figure S14****. ^1^H NMR spectrum of* ***MIP*** *in D_2_O. X indicates peaks from residual solvent.*

***
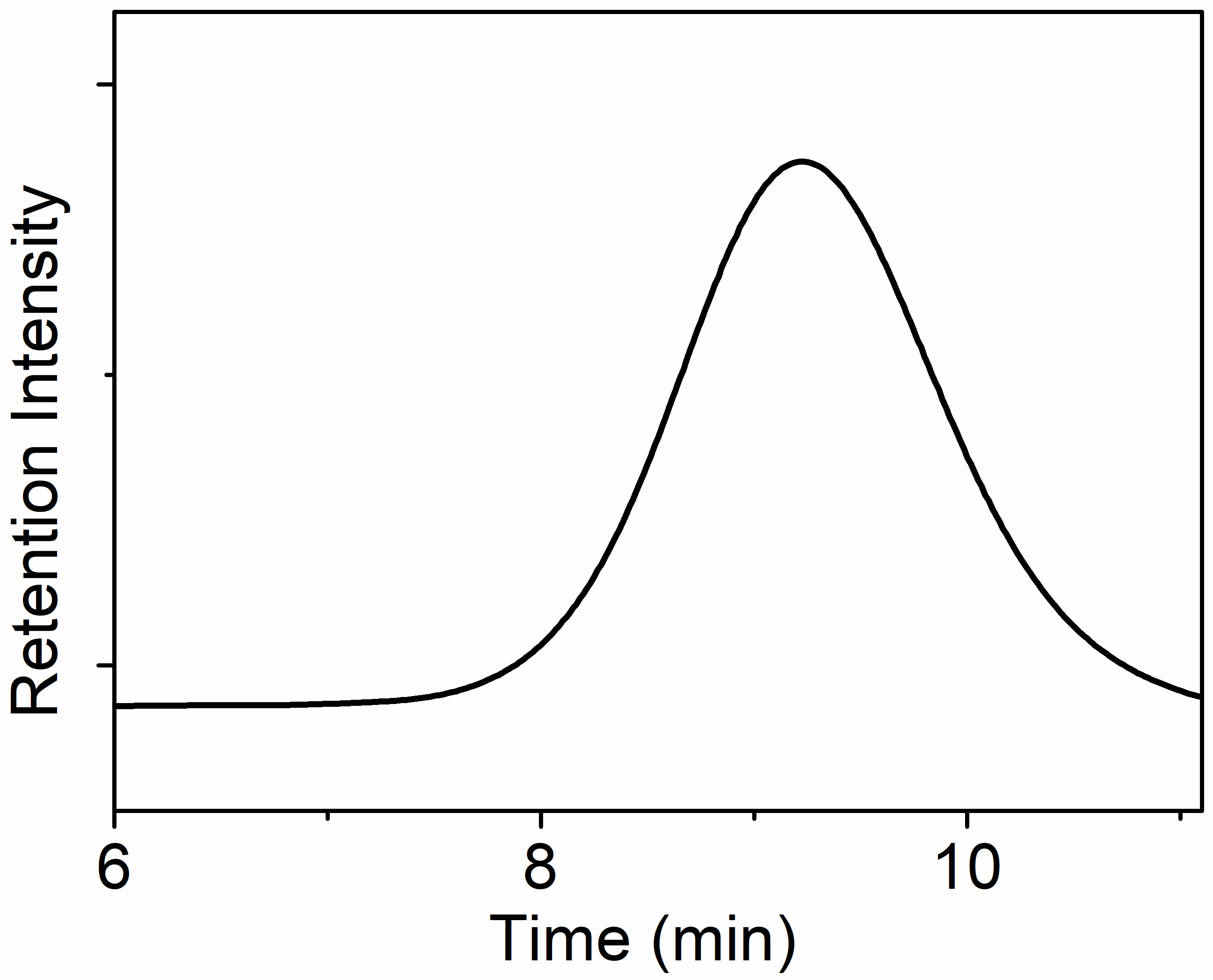
***

***\Figure S15****. SEC traces of* ***MIP*** *in water.*

**3. References**

[1] C. L. Wardzala, A. M. Wood, D. M. Belnap, J. R. Kramer, *ACS Cent. Sci*. **2022**, *8*, 351.

[2] M. Wyszogrodzka, R. Haag, *Chem. Eur. J.* **2008,** *14*, 9202.

[3] T. M. Legge, A. T. Slark, S. Perrier, *Macromolecules* **2007**, *40*, 2318.

[4] R. Bej, C. Nie, K. Ludwig, V. Ahmadi, J. Trimpert, J. M. Adler, T. L. Povolotsky, K. Achazi, M. Kagelmacher, R. M. Vidal, J. Dernedde, B. B. Kaufer, R. Haag, *Angew. Chem. Int. Ed.* **2023**, e202304010.

[5] R. Bej, A. Ghosh, J. Sarkar, B. B. Das, S. Ghosh, *ChemBioChem* **2020**, *21,* 2921.

[6] M. Gradzielski, R. Dalgliesh, N. Mahmoudi, R. F. Schmidt, H. Rulff, Y. Sun, **2022,** https://doi.org/10.5286/ISIS.E.RB2220343.

[7] O. Arnold, J. C. Bilheux, J. M. Borreguero, A. Buts, S. I. Campbell, L. Chapon, M. Doucet, N. Draper, R. Ferraz Leal, M. A. Gigg, V. E. Lynch, A. Markvardsen, D. J. Mikkelson, R. L. Mikkelson, R. Miller, K. Palmen, P. Parker, G. Passos, T. G. Perring, P. F. Peterson, S. Ren, M. A. Reuter, A. T. Savici, J. W. Taylor, R. J. Taylor, R. Tolchenov, W. Zhou, J. Zikovsky, *Nucl. Instrum. Methods Phys. Res. A* **2014,** *764*, 156.

[8] S.-H. Chen, T.-L. Lin, *Methods Exp. Phys.* **1987,** *23*, 489.

[9] G. D. Wignall, F. S. Bates, *J. Appl. Crystallogr.* **1987,** *20*, 28.
